# Neutrophil Mobilization Triggers Microglial Functional Change to Exacerbate Cerebral Ischemia‐Reperfusion Injury

**DOI:** 10.1002/advs.202503722

**Published:** 2025-06-25

**Authors:** Huijuan Jin, Zhifang Li, Senwei Tan, Qinghui Xiao, Qingcan Li, Jiao Ye, Yifan Zhou, Yan Wan, Qiang Liu, Bijoy K Menon, Bo Hu

**Affiliations:** ^1^ Department of Neurology Union Hospital Tongji Medical College Huazhong University of Science and Technology Wuhan 430022 China; ^2^ Department of Neurology Tianjin Neurological Institute Tianjin Medical University General Hospital Tianjin 300052 China; ^3^ Department of Community Health Sciences Department of Clinical Neurosciences Department of Radiology and Hotchkiss Brain Institute Cumming School of Medicine University of Calgary Calgary AB T2N 1N4 Canada

**Keywords:** ischemia‐reperfusion injury, microglial phagocytosis, microglial pyroptosis, neutrophil

## Abstract

Acute ischemic stroke is a leading cause of mortality and disability worldwide. Neuroinflammation following ischemia‐reperfusion plays a critical role in the disease's pathogenesis. Neutrophil aggregation and clearance within the brain parenchyma influence neuroinflammatory damage during ischemic stroke. Microglia‐mediated phagocytosis plays a pivotal role in mitigating neuroinflammation and promoting brain parenchyma recovery. However, the mechanisms underlying the cross‐talk between neutrophils and microglia remain poorly understood. Here, this study demonstrates that neutrophils can trigger microglial functional change to inhibit microglial phagocytosis and promote pyroptosis, which is regulated by neutrophil‐derived myeloid‐related protein 14. Additionally, interleukin‐1*β* released by pyroptotic microglia further upregulates myeloid‐related protein 14 expression and facilitates neutrophil mobilization from the bone marrow, establishing a self‐sustaining inflammatory loop. Therefore, neutrophils accumulate in the brain parenchyma and further exacerbate microglial neuroinflammation in the ischemic brain. These findings reveal a previously unknown interaction between neutrophils and microglia after acute ischemic stroke and suggest that targeting myeloid‐related protein 14 may provide a novel therapeutic strategy for ischemic stroke therapy.

## Graphical abstract

These study underscores the pivotal role of neutrophil‐derived myeloid‐related protein 14 (MRP14) in the pathophysiology of acute ischemic stroke (AIS). MRP14 not only disrupts mitochondrial function, thereby inhibiting microglial phagocytosis of neutrophils, but also activates microglial pyroptosis, exacerbating neuroinflammation following AIS. Targeting MRP14 could offer a promising therapeutic strategy to mitigate neuroinflammation and improve outcomes for patients with AIS.

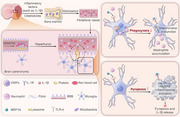

## 1 Introduction

Acute ischemic stroke (AIS) is a leading cause of mortality and disability worldwide.^[^ ^1^ ^]^ Extensive research has demonstrated that neuroinflammation, driven by activated immune cells following ischemia‐reperfusion, plays a critical role in the pathogenesis of ischemic stroke, contributing to secondary brain injury.^[^ ^2^, ^3^ ^]^ Neutrophils, as key neuroinflammatory mediators, are among the first peripheral immune cells to infiltrate the brain, arriving within minutes to hours after stroke onset. Their rapid mobilization from the bone marrow and spleen is triggered by chemokines and inflammatory cytokines released by ischemic brain tissue.^[^ ^4^ ^]^ Within the ischemic microenvironment, neutrophils become hyperactivated and release proteolytic enzymes, which exacerbate the recruitment of additional peripheral immune cells and the activation of brain‐resident immune cells; this cascade further amplifies neuroinflammation.^[^ ^4^, ^5^, ^6^ ^]^ Previous studies, including those from our laboratory, have demonstrated that elevated peripheral neutrophil counts during the early stages of AIS are associated with increased cerebral edema, larger infarct volumes, and poorer prognosis.^[^ ^7^, ^8^ ^]^ Although experimental strategies aimed at reducing neutrophil migration and infiltration have shown promise in limiting brain injury, clinical trials have not yielded encouraging results,^[^ ^9^ ^]^ primarily due to insufficient focus on the clearance of infiltrating neutrophils within ischemic brain tissue.

Microglia, as the principal resident immune cells of the central nervous system, serve as primary responders following ischemic stroke. Microglia‐mediated phagocytosis plays a pivotal role in mitigating neuroinflammation and aiding brain parenchymal recovery. By engulfing apoptotic cells before they undergo secondary necrosis, which would otherwise release harmful intracellular components, microglia help modulate the overall inflammatory response.^[^ ^10^ ^]^ Research has shown that microglia can actively phagocytize both apoptotic and viable neutrophils recruited to the ischemic brain.^[^ ^11^ ^]^ However, ischemia significantly impairs microglial phagocytic function due to energy depletion and cellular atrophy, resulting in increased neutrophil accumulation and exacerbation of brain injury.^[^ ^10^, ^11^ ^]^ Therefore, enhancing microglial phagocytosis of neutrophils presents a promising therapeutic strategy for reducing brain injury and improving outcomes in ischemic stroke.

This study found novel interactions between neutrophils and microglia in both peripheral and intracranial immune responses after ischemic stroke. Our findings revealed that neutrophils can trigger microglial functional change to inhibit their phagocytic activity and promote pyroptosis, which is regulated by neutrophil‐derived myeloid‐related protein 14 (MRP14, also known as S100A9). In turn, interleukin‐1*β* (IL‐1*β*), released by pyroptotic microglia, further stimulates neutrophil mobilization from the bone marrow and upregulates MRP14 expression, creating a self‐sustaining inflammatory loop. This accumulation of neutrophils and pyroptotic microglia amplifies the inflammatory cascade, ultimately exacerbating blood‐brain barrier (BBB) disruption in ischemic stroke.

## 2 Results

### 2.1 Neutrophil Mobilization was Associated with Cerebral Edema and Microglial Function Changes after AIS

**Figure 1:**
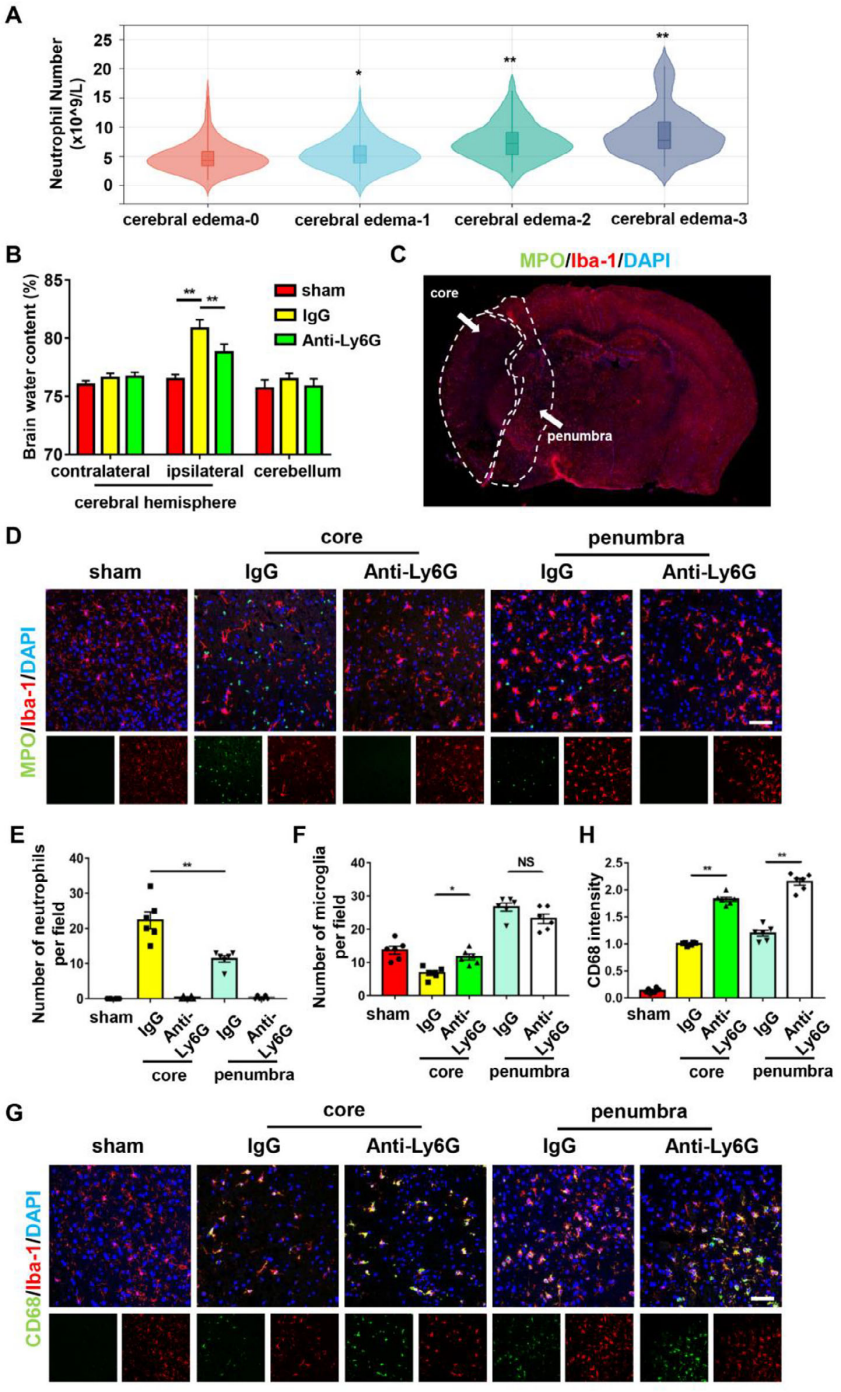
Neutrophil mobilization was associated with cerebral edema and microglial function changes after AIS. A) The number of peripheral neutrophils in patients with AIS with different cerebral edema grades. B) Brain water content of the contralateral cerebral hemisphere, ipsilateral cerebral hemisphere and cerebellum in the tMCAO mice after IgG or Anti‐Ly6G treatment (n = 6/group). C) Illustration of the penumbra and ischemic core in tMCAO mice. D‐F) The number of neutrophils and microglia in the penumbra and ischemic core of tMCAO mice after IgG or Anti‐Ly6G treatment (n = 6/group). G,H) Immunofluorescence staining showing the expression of CD68 in microglia in the penumbra and ischemic core of the tMCAO mice after IgG or Anti‐Ly6G treatment (n = 6/group). Data are presented as means ± SEM; one‐way ANOVA, two‐way ANOVA. **P < 0.01. Scale bar: 100 µm. AIS, acute ischemic stroke; tMCAO, transient middle cerebral artery occlusion.

A total of 414 AIS patients, who were consecutively enrolled from the Multicenter Clinical Trial of Revascularization Treatment for Acute Ischemic Stroke (TRAIS) cohort between January 1, 2019, and December 30, 2021, were analyzed to assess the association between neutrophil counts and cerebral edema severity (clinical characteristics are shown in Table S1, Supporting Information). As depicted in **Figure** **1**A, peripheral neutrophil counts measured within 24 h of stroke onset demonstrated a positive correlation with cerebral edema grade. To further validate this association, we injected mice with an anti‐Ly6G antibody to deplete neutrophils before undergoing transient middle cerebral artery occlusion (tMCAO). Compared with vehicle group, the inhibition of neutrophils significantly reduced the ipsilateral hemisphere brain water content, indicating the alleviation of cerebral edema (Figure 1B).

Next, we investigated the mechanisms by which neutrophils contribute to cerebral edema. Previous studies have demonstrated that, upon infiltrating the brain parenchyma, neutrophils interact with microglia and modulate their function following AIS. By performing double‐staining for neutrophils and microglia, we observed a higher density of neutrophils and a lower number of microglia in the ischemic core relative to the penumbra (Figure 1C‐F). Microglia within the core also exhibited signs of dystrophy and cell loss. Interestingly, neutrophil depletion led to an increase in microglial numbers in the ischemic core (Figure 1C‐F), suggesting a potential role for neutrophils in modulating microglial survival. In addition, we found three positional relationships between neutrophils and microglia in the ischemic brain: free, touching, and surrounded states (Figure S1A,B, Supporting Information). The latter two patterns are indicative of microglial phagocytosis of neutrophils. Assessing phagocytic function, we found that neutrophil depletion significantly increased CD68 intensity in microglia in both the core and ischemic penumbra (Figure 1G,H), suggesting that neutrophils also influence microglial phagocytosis. Collectively, these results delineated a potential interaction between neutrophils and microglia that may contribute to the pathogenesis of cerebral edema after AIS.

### 2.2 Neutrophil MRP14 Expression is Elevated in Patients with AIS Exhibiting Severe Cerebral Edema

**Figure 2:**
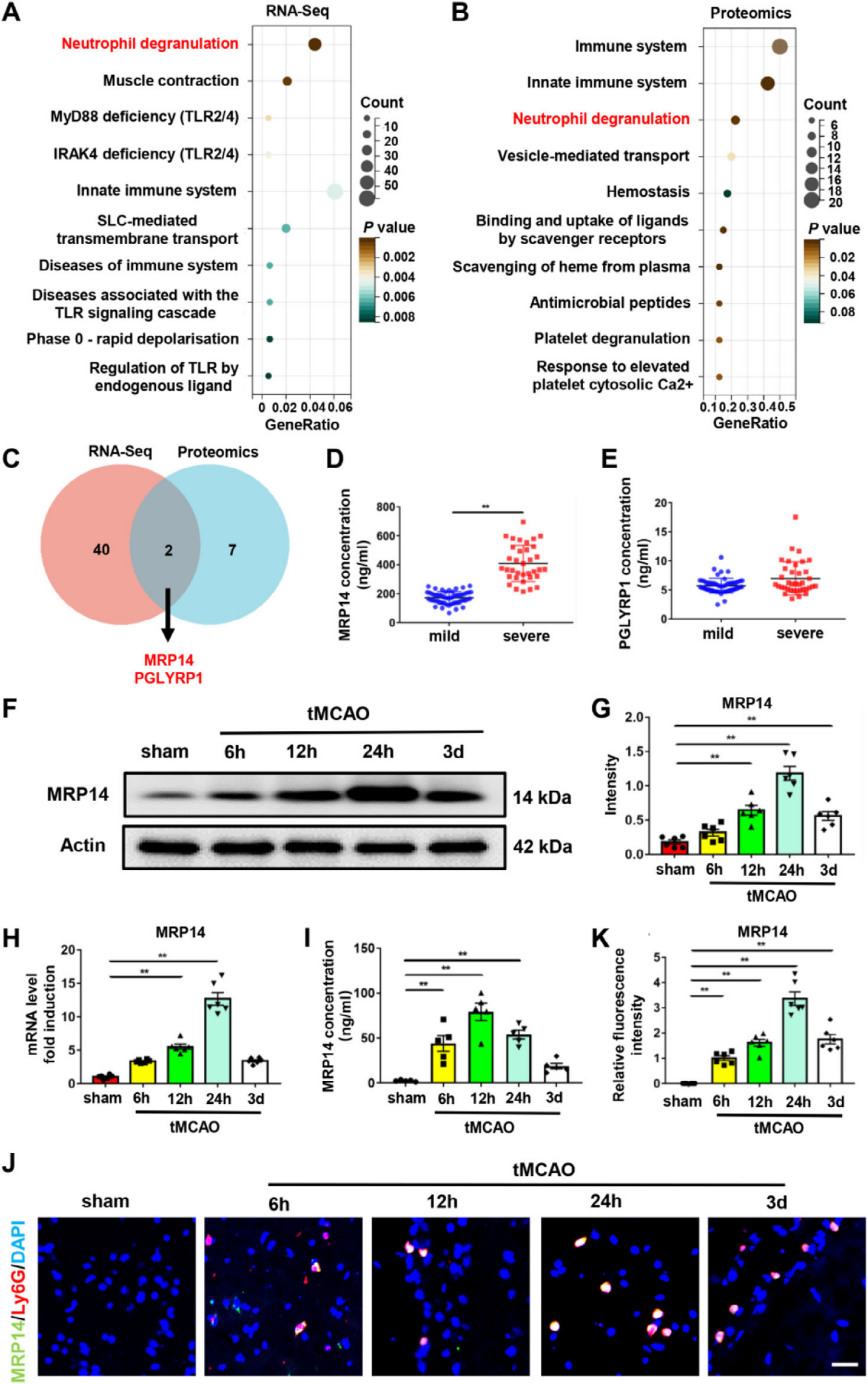
MRP14 expression is elevated in patients with AIS exhibiting severe cerebral edema and in the tMCAO mice. A) The REACTOME pathway enrichment analysis of differentially expressed genes in the peripheral neutrophils of patients with AIS exhibiting either mild or severe cerebral edema in the RNA‐sequencing. B) The REACTOME pathway enrichment analysis of differentially expressed proteins in the plasma proteomics of patients with AIS exhibiting either mild or severe cerebral edema. C) The Venn diagram showing the overlapped genes between the “Neutrophil degranulation” pathway in the RNA‐sequencing and proteomics. D,E) The ELISA test of MRP14 and PGLYRP1 level in the plasma of patients with AIS exhibiting either mild or severe cerebral edema (mild, n = 53; severe, n = 35). F,G) Western blot analysis of the MRP14 protein level in the ischemic brain of the tMCAO mice at different time points (n = 6/group). H) The mRNA level of MRP14 in the ischemic brain of the tMCAO mice at different time points (n = 6/group). I) Quantification of the plasma MRP14 level by ELISA assay of the tMCAO mice at different time points (n = 6/group). J,K) Immunofluorescence staining showing the MRP14^+^ neutrophils in the ischemic brain of the tMCAO mice at different time points (n = 6/group). Data are presented as means ± SEM; unpaired t‐test, one‐way ANOVA. *P < 0.05, **P < 0.01. Scale bar: 25 µm. AIS, acute ischemic stroke; tMCAO, transient middle cerebral artery occlusion; ELISA, enzyme‐linked immunosorbent assay.

To determine the role of neutrophils in cerebral edema and microglial function, peripheral neutrophils were collected from six patients with AIS exhibiting either mild or severe cerebral edema. After matching for clinical characteristics, RNA sequencing was performed. A total of 1319 differentially expressed genes (DEGs) were identified between the mild and severe cerebral edema groups (|Log2FC| > 1). REACTOME analysis of these DEGs identified “Neutrophil degranulation” as the most significantly enriched pathway (**Figure** **2**A). Proteomic analysis was conducted using plasma samples from the aforementioned two groups. This analysis revealed 63 differentially expressed proteins between the two groups (|Log2FC| > 1), with REACTOME enrichment highlighting “Immune system”, “Innate immune system”, and “Neutrophil degranulation” as the top three enriched pathways (Figure 2B). These findings highlight the critical role of neutrophil‐driven inflammation in cerebral edema. Among the DEGs and differentially expressed proteins, MRP14 and peptidoglycan recognition protein 1 (PGLYRP1) emerged as overlapping genes within the “Neutrophil degranulation” pathway identified in both transcriptomic and proteomic analyses (Figure 2C). Upon expanding the sample size, plasma levels of MRP14 were significantly higher in patients with severe cerebral edema compared with those with mild edema (Figure 2D), whereas PGLYRP1 levels did not differ significantly between the groups (Figure 2E). These results suggest that elevated plasma MRP14 levels are associated with severe cerebral edema in AIS.

### 2.3 Neutrophil MRP14 Expression is Increased in the Ischemic Brain of tMCAO Mice

We further validated MRP14 expression after ischemic stroke using two independent datasets. The first was our own RNA sequencing dataset from peripheral neutrophils isolated from sham and tMCAO mice 24 h after stroke (Figure S2A,B, Supporting Information). The second dataset (GSE174440) consisted of single‐cell RNA sequencing (scRNA‐seq) data from sorted blood myeloid cells of mice at steady state and 24 h after tMCAO (Figure S2C, Supporting Information). Both datasets showed elevated neutrophil MRP14 expression following ischemic stroke (Figure S2B,C, Supporting Information).

Next, we collected ischemic brain tissues and plasma from tMCAO mice at various time points to assess changes in MRP14 expression. Both protein and mRNA levels of MRP14 in the ischemic brain started to rise at 6 h post‐tMCAO, peaking at 24 h (Figure 2F‐H). In plasma, MRP14 levels started to increase at 6 h, reaching a peak at 12 h post‐tMCAO (Figure 2I). To further determine the cellular source of MRP14 in the ischemic brain, double immunostaining was performed using the neutrophil marker Ly6G and the microglial marker Iba‐1. The results demonstrated that MRP14 predominantly co‐localized with neutrophils (Figure 2J,K), with minimal expression observed in microglia at 3 days post‐tMCAO (Figure S3, Supporting Information). These results provide strong evidence that neutrophils are the primary source of elevated MRP14 in the ischemic brain following tMCAO.

### 2.4 Both paquinimod Treatment and MRP14 Knockout Attenuate BBB Disruption after Ischemic Stroke

**Figure 3:**
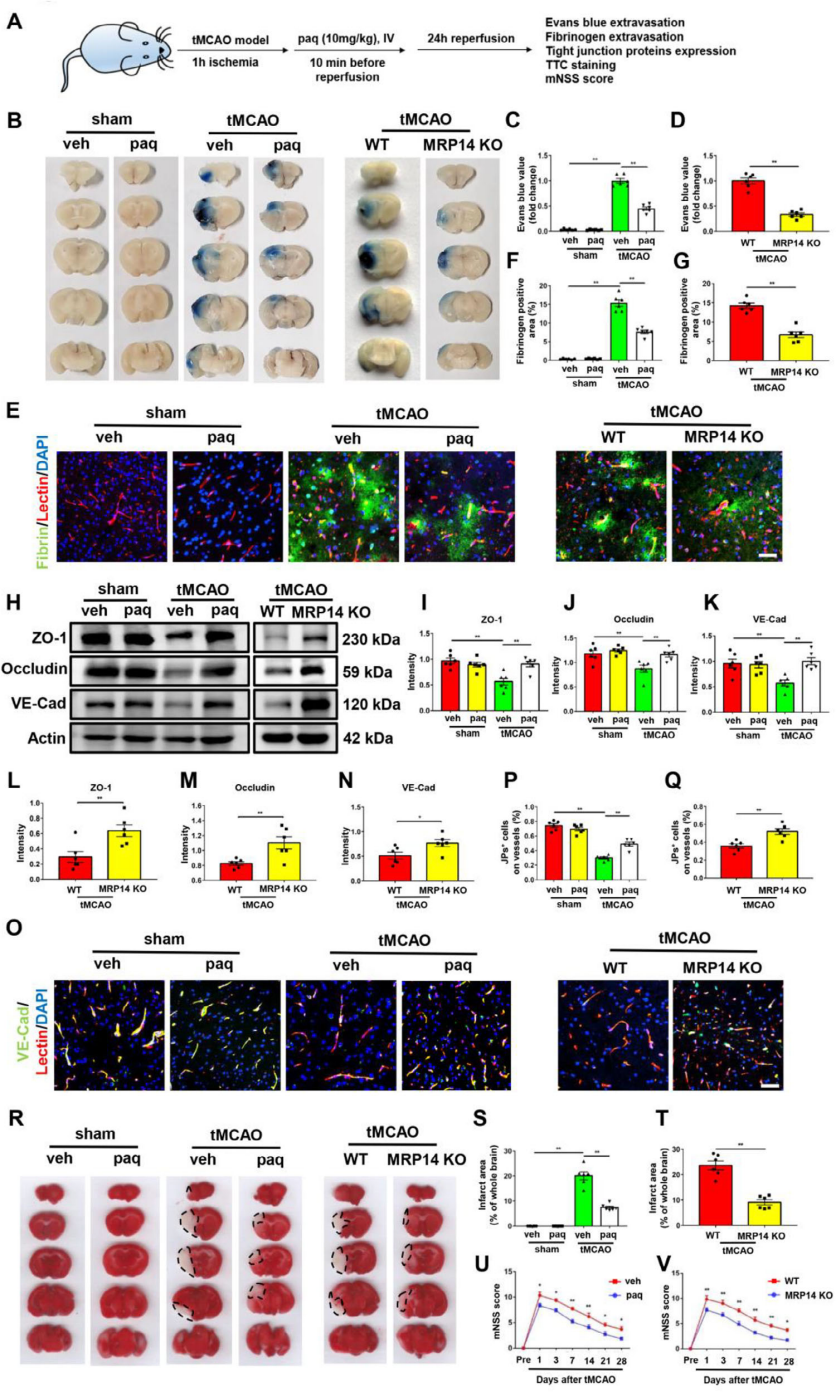
Paquinimod treatment or MRP14 knockout attenuates BBB disruption after ischemic stroke. A) The illustration of the experimental schedule. The mice were first subjected to tMCAO procedure for 1‐h ischemia and paquinimod (10 mg kg^−1^) was injected intravenously 10 min before reperfusion. The subsequent experiments were performed 24 h after reperfusion. B‐D) The Evans blue extravasation assay of the indicated groups (n = 6/group). E‐G) Immunofluorescence staining exhibiting the fibrinogen (green) leakage in the ischemic brain of the indicated groups (n = 6/group). H‐N) Western blot analysis of the expression of tight junction proteins, including ZO‐1, Occludin and VE‐Cadherin in the ischemic brain of the indicated groups (n = 6/group). O‐Q) Immunofluorescence staining showing the expression of VE‐Cadherin (green) on the blood vessels (red) in the ischemic brain of the indicated groups (n = 6/group). R‐T) The TTC staining showing the infarction area in the indicated groups (n = 6/group). U,V) The mNSS scores showing the neurological deficits in the indicated groups (n = 6). Data are presented as means ± SEM; unpaired t‐test, one‐way ANOVA. *P < 0.05, **P < 0.01. Scale bar: 50 µm. BBB, blood brain barrier; tMCAO, transient middle cerebral artery occlusion; TTC, tri‐phenyl tetrazolium chloride; mNSS, Modified Neurological Severity Score; veh, vehicle; paq, paquinimod.

The disruption of BBB integrity occurs 24–72 h post‐ischemic stroke,^[^ ^12^ ^]^ corresponding to elevated intracranial levels of MRP14. To investigate whether increased MRP14 contributes to pathological BBB leakage, the MRP14 inhibitor paquinimod was intravenously administered into mice, to inhibit extracellular MRP14 function. BBB integrity and infarct area were assessed 1 day after tMCAO, and neurological deficits were assessed 1, 3, 7, 14, 21, and 28 days after tMCAO (**Figure** **3**A). Mice subjected to tMCAO exhibited enhanced extravasation of Evans blue and fibrinogen, indicating BBB disruption, while paquinimod treatment significantly attenuated this leakage (Figure 3B,C,E,F). Additionally, expression of tight junction proteins (ZO‐1, Occludin, VE‐Cadherin) was reduced in tMCAO mice, which was counteracted by paquinimod treatment (Figure 3H‐K,O,P). Paquinimod also mitigated infarct area and neurological outcomes in these mice (Figure 3R,S,U). These results suggest that MRP14 inhibition can alleviate BBB breakdown following ischemic stroke. To further validate MRP14's role, MRP14 knockout (KO) mice were subjected to tMCAO (Figure S4A‐D, Supporting Information). Compared with wild type (WT) mice, MRP14 KO mice exhibited significantly reduced BBB leakage, as evidenced by Evans blue and fibrinogen extravasation assays 24 h after tMCAO (Figure 3B,D,E,G). These mice also showed restored tight junction protein expression (Figure 3H,L‐O,Q), decreased infarct area and improved neurological outcomes (Figure 3R,T,V). These results underscore the pivotal role of MRP14 in mediating BBB disruption post‐stroke.

To specifically elucidate the role of neutrophil‐derived MRP14 in BBB damage, neutrophils were isolated from the bone marrow of WT or MRP14 KO mice, labeled with Molday ION Rhodamine B (MIRB), and transferred into MRP14 KO mice via tail vein injection prior to tMCAO (Figure S5A, Supporting Information). MIRB‐labeled neutrophils were detected in the ischemic brain 24 h post‐tMCAO (Figure S5B, Supporting Information), confirming successful neutrophil transfer. Mice receiving WT neutrophils exhibited significantly greater BBB leakage, as evidenced by both Evans blue and fibrinogen assays (Figure S5C‐F, Supporting Information), than those receiving MRP14 KO neutrophils. Furthermore, WT neutrophil transfer led to decreased tight junction protein expression (Figure S5G‐L, Supporting Information), larger infarct areas, and worsened neurological deficits (Figure S5M‐O, Supporting Information). These results strongly indicate that neutrophil‐derived MRP14 is critical to BBB disruption following ischemic stroke.

### 2.5 MRP14 Triggers Microglial Functional Change after Ischemic Stroke

Considering that MRP14, a member of damage‐related molecular patterns, triggers pro‐inflammatory immune responses,^[^ ^13^ ^]^ and that microglia and astrocytes serve as the primary immune cells in the brain, we isolated neutrophils from WT or MRP14 KO tMCAO mice, and then co‐cultured them with oxygen‐glucose deprivation/reoxygenation (OGD/R)‐treated primary microglia or astrocytes for 24 h. The supernatants were then added into a Transwell chamber, with endothelial bEnd.3 cells seeded in the upper compartment (Figure S6A, Supporting Information). As shown in Figure S6B,C (Supporting Information), supernatants from WT neutrophils reduced transendothelial electrical resistance (TEER) value and increased FITC‐dextran permeability in endothelial cells. These effects were alleviated by supernatants from MRP14 KO neutrophils, indicating that neutrophils‐derived MRP14 alone could lead to endothelial damage. Notably, these effects were amplified when endothelial cells were exposed to supernatants from WT neutrophil‐microglia co‐cultures compared with those from WT neutrophil‐astrocyte co‐cultures (Figure S6B,C, Supporting Information). Furthermore, supernatants from MRP14 KO neutrophil‐microglia co‐cultures attenuated BBB leakage compared with those from WT counterparts (Figure S6B,C, Supporting Information). Previous studies have indicated that MRP14 binds with high affinity to Toll‐like receptor 4 (TLR4) and the receptor for advanced glycation end‐products (RAGE),^[^ ^14^, ^15^ ^]^ which facilitate MRP14 signaling. We further found that only TLR4 inhibitor resatorvid, not RAGE inhibitor FPS‐ZM1, significantly reversed the effect of WT neutrophil‐microglia co‐cultures supernatants in the in vitro BBB model (Figure S6D,E, Supporting Information). Together, these results suggest that MRP14 could induce more pronounced endothelial cell damage by stimulating microglia, which is mediated by TLR4 signaling.

**Figure 4:**
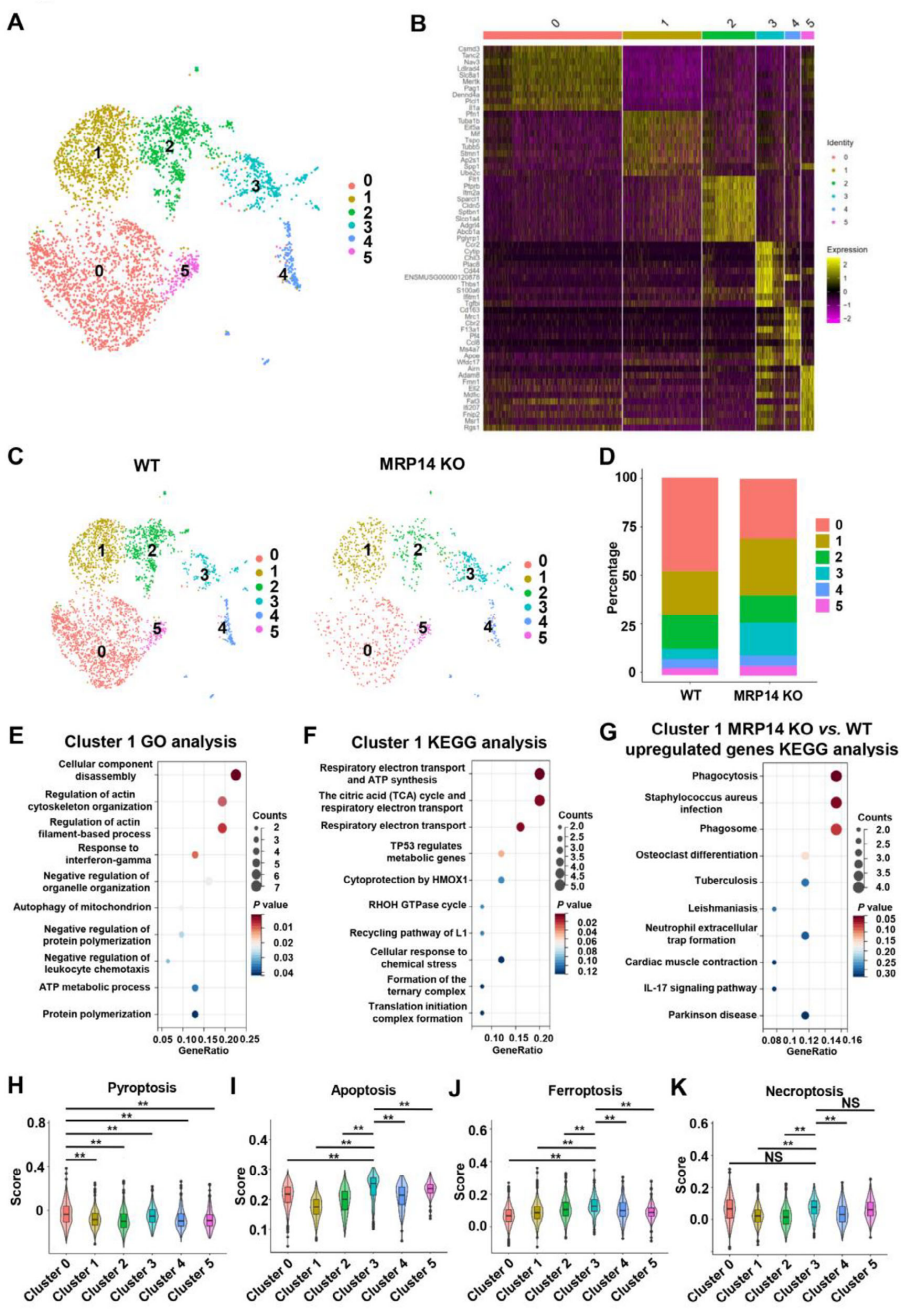
Characteristics of microglia in the MRP14 KO mice 24 h after tMCAO. A) The UMAP plot displaying the 6 microglial clusters. B) Heatmap showing the top 10 signature genes in each cluster. C) The distribution of microglia in the WT and MRP14 KO groups in the UMAP plot. D) Bar graph showing the percentage of each microglial cluster in the WT and MRP14 KO groups. E) The top 10 enriched GO terms of biologic process of cluster 1. F) The top 10 enriched KEGG terms of cluster 1. G) The top 10 enriched KEGG terms of differentially expressed genes between the WT and MRP14 KO groups in the cluster 1. H‐K) The violin plots showing the AUC scores of different death pathway activities of each cluster. Data are presented as means ± SEM; one‐way ANOVA. **P < 0.01. tMCAO, transient middle cerebral artery occlusion; WT, wild type; KO, knockout; UMAP, Uniform Manifold Approximation and Projection; GO, Gene Ontology; KEGG, Kyoto Encyclopedia of Genes and Genomes; AUC, Area Under Curve.

To further elucidate the underlying mechanisms, the scRNA‐seq analysis was conducted on microglia isolated from ischemic brain tissue of WT and MRP14 KO tMCAO mice. The cells were integrated using the “harmony” package in R. Microglia were identified by the markers “Trem2”, “Tmem119”, and “Siglech”. The uniform manifold approximation and projection (UMAP) visualization revealed six distinct microglial subpopulations (**Figure** **4**A), and a heatmap displayed the relative expression of the top 10 signature genes within each cluster (Figure 4B). Notably, clusters 0 and 1 were the dominant subpopulations. Compared with the WT group, the percentage of cluster 0 significantly reduced and cluster 1 markedly increased in the MRP14 KO group (Figure 4C,D). Gene ontology (GO) and Kyoto encyclopedia of genes and genomes (KEGG) pathway analyses linked cluster 1 to microglial motility and energy metabolism (Figure 4E,F). Enrichment analysis of DEGs upregulated in cluster 1 between WT and MRP14 KO groups identified “Phagocytosis” and “Phagosome” as top pathways (Figure 4G), suggesting an association of cluster 1 microglia with phagocytic function. Given the elevated proportion of this cluster in MRP14 KO mice, we assume that neutrophil‐derived MRP14 may impair microglial phagocytosis following AIS. In addition to affecting phagocytosis, infiltrating neutrophils can also influence microglial viability. Previous results have demonstrated an inverse correlation between microglial counts and the number of infiltrating neutrophils in the ischemic brain (Figure 1D‐F). To further explore this relationship, area under the curve scores of cell death pathway activities were calculated for identified microglial clusters (clusters 0–5). Cluster 0, which proportion was most significantly reduced in the MRP14 KO group, exhibited the highest activity in the pyroptosis pathway (Figure 4H‐K), suggesting that MRP14 may contribute to microglial pyroptosis.

To further investigate the transformation among microglial clusters, a pseudotime trajectory analysis was performed (Figure S7A, Supporting Information). We found that cluster 1, phagocytosis‐related microglia, was located at the start and middle stages of the developmental trajectory, while cluster 0, pyroptosis‐related microglia, appeared at the end stage (Figure S7B, Supporting Information). This suggested a potential transition from cluster 1 to cluster 0 (Figure S7C, Supporting Information). In line with this observation, the expression of phagocytosis‐related genes was decreased and pyroptosis‐related genes was increased along the pseudotime trajectory (Figure S7D,E, Supporting Information).

### 2.6 MRP14 Inhibits Microglial Phagocytosis of Neutrophils and Promotes Microglial Pyroptosis after Ischemic Stroke

**Figure 5:**
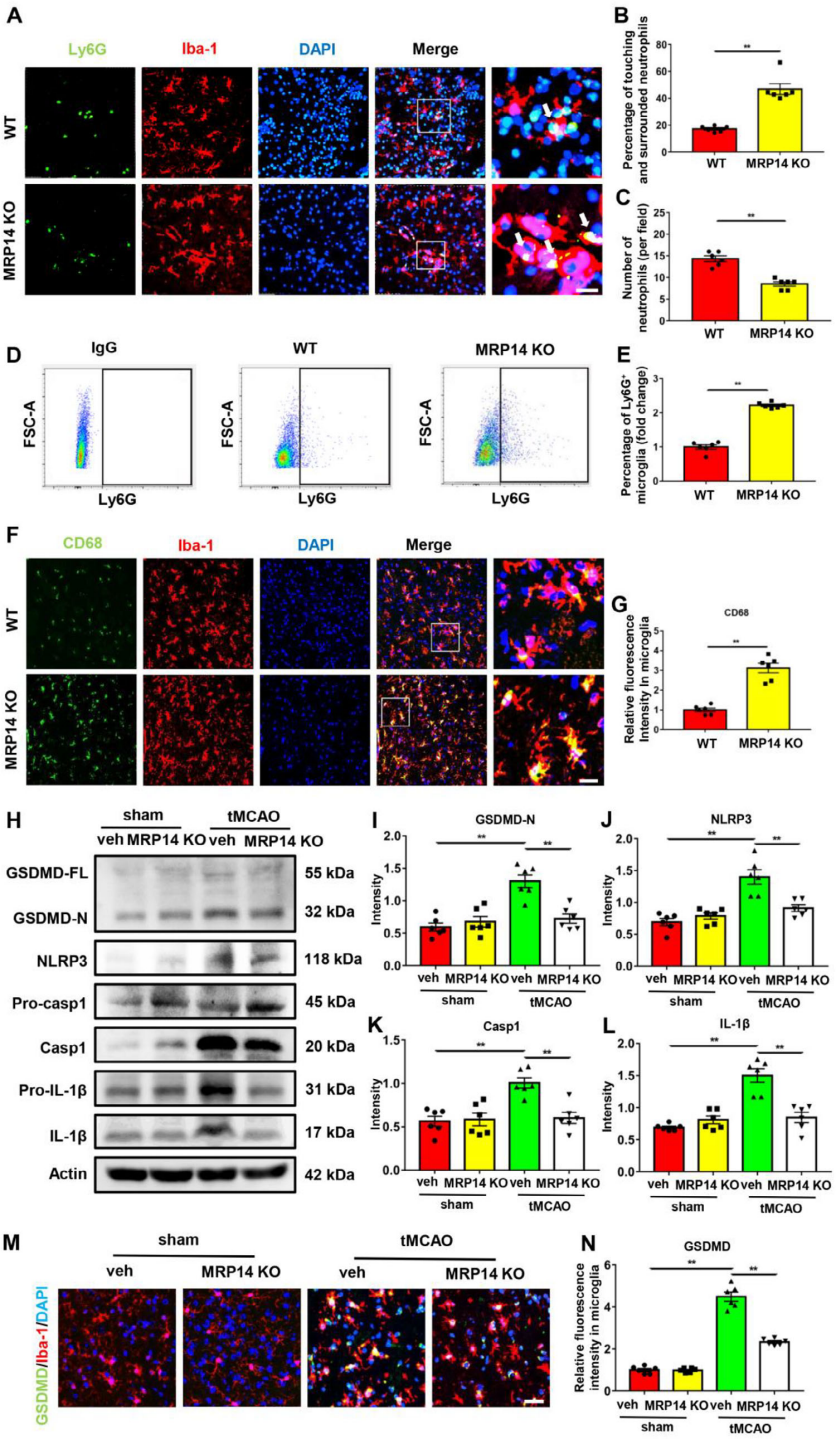
MRP14 inhibits microglia to phagocytize neutrophils and promotes microglial pyroptosis after ischemic stroke. A,B) Immunofluorescence staining showing the percentage of neutrophils (green) touching or surrounded by microglia (red) in the ischemic brain of WT and MRP14 KO groups (n = 6/group). The white arrow indicates neutrophils touching or surrounded by microglia. C) The neutrophil numbers per field in the ischemic brain of WT and MRP14 KO groups (n = 6/group). D,E) The flow cytometry analysis showing the percentage of Ly6G^+^ microglia in the ischemic brain of the tMCAO mice from the WT and MRP14 KO groups (n = 6/group). IgG was used as a negative control. F,G) Immunofluorescence staining showing the expression of CD68 (green) in the microglia (red) in the ischemic brain of WT and MRP14 KO groups (n = 6/group). H‐L) Western blot analysis of the expression of GSDMD, NLRP3, pro‐caspase 1, caspase 1, pro‐IL‐1*β* and IL‐1*β* in the ischemic brain of WT or MRP14 KO mice 24 h after tMCAO (n = 6/group). M,N) Immunofluorescence staining showing the expression of GSDMD (green) in the microglia (red) in the ischemic brain of WT or MRP14 KO mice 24 h after tMCAO (n = 6/group). Data are presented as means ± SEM; Mann‐Whitney test, unpaired t‐test, one‐way ANOVA. **P < 0.01. Scale bar: 25 µm (A and F); 50 µm (M). tMCAO, transient middle cerebral artery occlusion; WT, wild type; KO, knockout; veh, vehicle; paq, paquinimod; GSDMD, gasdermin D; NLRP3, NOD‐like receptor family, pyrin domain containing 3; IL‐1*β*, interleukin‐1*β*; Casp1, caspase 1.

Previous results have suggested that activated microglia can engulf neutrophils recruited to the ischemic brain (Figure S1A,B, Supporting Information). Thus, this study further investigated whether MRP14 regulates microglial phagocytosis of neutrophils. Microglial phagocytosis of neutrophils was quantified by calculating the percentage of neutrophils in touching and surrounded states in vivo. As shown in **Figure** **5**A,B, MRP14 KO mice exhibited a significantly higher percentage of neutrophils touching and surrounded by microglia compared with WT mice, 24 h post‐tMCAO. Additionally, the overall neutrophil count was reduced in the MRP14 KO group (Figure 5C). In addition, we also used flow cytometry to quantify the ability of microglia to engulf neutrophils. First, we labeled microglia with CD45 and CD11b in the ischemic brain cells isolated from WT and MRP14 KO mice 24 h post‐tMCAO. Then, the cells were permeabilized and intracellularly stained with neutrophil marker Ly6G. Finally, the percentage of Ly6G^+^ microglia, which indicates the microglia that have phagocytosed neutrophils, was analyzed using flow cytometry (Figure S8, Supporting Information). Compared with the WT group, MRP14 KO mice exhibited a significantly higher percentage of Ly6G^+^ microglia, suggesting an increase in phagocytosis‐related microglia (Figure 5D,E). To further assess changes in phagocytic capacity, the expression of CD68, a marker of active phagocytosis, was measured in microglia. Compared with WT mice, MRP14 KO mice showed enhanced microglial CD68 fluorescence intensity 24 h after tMCAO (Figure 5F,G). Together, these results suggested that MRP14 prevents microglial phagocytosis of neutrophils after ischemic stroke.

The role of MRP14 in promoting microglial pyroptosis was further assessed in vivo. Elevated expression of pyroptosis‐associated proteins, including GSDMD, NLRP3, caspase‐1 and IL‐1*β*, was observed in the ischemic brain tissue of tMCAO mice compared with the sham group (Figure 5H‐L). However, MRP14 deficiency significantly reversed these increases (Figure 5H‐L). Immunofluorescence staining further revealed a marked increase in GSDMD fluorescence intensity in microglia 24 h post‐tMCAO (Figure 5M,N); this increase was similarly attenuated by MRP14 deficiency (Figure 5M,N). These results suggest that MRP14 may be involved in activating NLRP3 inflammasome‐mediated microglial pyroptosis following ischemic stroke in vivo.

### 2.7 MRP14 Impairs Microglial Phagocytosis of Neutrophils by Triggering Mitochondrial Dysfunction via TLR4 Receptor In Vitro

The effect of MRP14 on microglial phagocytic activity was further explored in vitro. Neutrophils were isolated from the tMCAO mice, and microglia were exposed to OGD/R followed by stimulation with recombinant MRP14. After 24 h, neutrophils and microglia were co‐cultured to assess the effect of MRP14 on microglial phagocytosis (Figure S9A, Supporting Information). As demonstrated in Figure S9B,C (Supporting Information), OGD/R‐treated microglia exhibited increased CD68 expression and enhanced neutrophil phagocytosis, both of which were significantly attenuated by recombinant MRP14 treatment. Additionally, Western blot analysis revealed elevated MPO expression in OGD/R‐treated microglia, an effect that was reversed by recombinant MRP14 treatment (Figure S9D,E, Supporting Information). We also investigated the role of TLR4 in mediating the effects of MRP14 on microglial phagocytosis. As shown in Figure S9B‐E (Supporting Information), inhibition of TLR4 with resatorvid markedly reduced the inhibitory effect of recombinant MRP14 on microglial phagocytosis of neutrophils, underscoring the key role of TLR4 in this process.

Efficient phagocytosis by microglia requires dynamic cytoskeletal reorganization, a process heavily dependent on adenosine triphosphatase (ATP).^[^ ^16^ ^]^ Prior studies have demonstrated that MRP14 induces mitochondrial dysfunction through TLR4 after myocardial infarction.^[^ ^17^ ^]^ Consistently, KEGG pathway analysis of cluster 1 signature genes identified enrichment in pathways related to the tricarboxylic acid cycle, respiratory electron transport, and ATP synthesis (Figure 4F). These findings support the hypothesis that MRP14 disrupts mitochondrial function in microglia, thereby compromising the energy supply necessary for phagocytosis. JC‐1 staining confirmed that OGD/R treatment significantly decreased the red/green fluorescence ratio in microglia, indicating mitochondrial membrane potential disruption, and recombined MRP14 treatment could further exacerbate the damage (Figure S9F,G, Supporting Information). However, this damage was mitigated by TLR4 inhibition with resatorvid. Similarly, ATP concentration in OGD/R‐treated microglia were decreased, with further reductions observed following recombined MRP14 treatment. These ATP deficits were also mitigated by resatorvid‐mediated TLR4 inhibition (Figure S9H, Supporting Information). Collectively, these results suggest that MRP14 may impair microglial phagocytosis of neutrophils by inducing mitochondrial dysfunction via the TLR4 receptor.

### 2.8 MRP14 Promotes Microglial NLRP3 Inflammasome Activation and Pyroptosis Through TLR4/NF‐κB Signaling In Vitro

The effect of MRP14 on microglial pyroptosis was also investigated in vitro. In primary microglia cultures, treatment with recombinant MRP14 alone did not induce cleavage of caspase‐1 or IL‐1*β*, as shown by Western blot analysis (Figure S10A‐C, Supporting Information). However, priming with recombinant MRP14 followed by ATP treatment, but not lipopolysaccharide (LPS), resulted in significant processing of caspase‐1 and IL‐1*β* (Figure S10A‐C, Supporting Information). These findings indicate that MRP14 functions as a priming signal, similar to LPS, in the induction of microglial pyroptosis. In subsequent mechanistic studies, primary microglia were treated with both recombinant MRP14 and ATP. Additionally, primary microglia were further treated with recombinant MRP14 and/or the TLR4 inhibitor resatorvid. The results demonstrated that recombinant MRP14 significantly upregulated the protein expression of GSDMD, NLRP3, caspase‐1 and IL‐1*β* in microglia (Figure S10D‐H, Supporting Information), and increased the release of IL‐1*β* into the supernatant (Figure S10I, Supporting Information). Resatorvid effectively blocked these effects, indicating that the pro‐inflammatory actions of MRP14 are mediated through the TLR4 pathway (Figure S10D‐I, Supporting Information).

To further elucidate the downstream mechanisms of MRP14‐mediated pyroptosis, key signaling pathways were examined. Prior studies have shown that TLR4 predominantly activates the NF‐κB signaling pathway to facilitate intracellular functions.^[^ ^18^ ^]^ Our findings revealed that MRP14 significantly enhanced the phosphorylation of IκB and p65, and this effect was abolished by TLR4 inhibition (Figure S11A‐D, Supporting Information). These results suggest that MRP14 regulates NF‐κB activation via TLR4. To confirm that MRP14 exerts its effects on microglia pyroptosis via the NF‐κB pathway, the NF‐κB inhibitor JSH‐23 was employed to suppress its activity. Notably, NF‐κB inhibition significantly diminished the MRP14‐induced upregulation of GSDMD, NLRP3, caspase‐1 and IL‐1*β*, as well as the release of IL‐1*β* into the supernatant (Figure S11E‐J, Supporting Information). Collectively, these results suggest that MRP14 promotes NLRP3 inflammasome‐associated pyroptosis in primary microglia through activation of the TLR4/NF‐κB signaling pathway.

### 2.9 Microglial TLR4 Knockout Reduces MRP14‐Induced Phagocytosis Impairment, NLRP3 Inflammasome‐Associated Pyroptosis, and BBB Breakdown In Vivo

**Figure 6:**
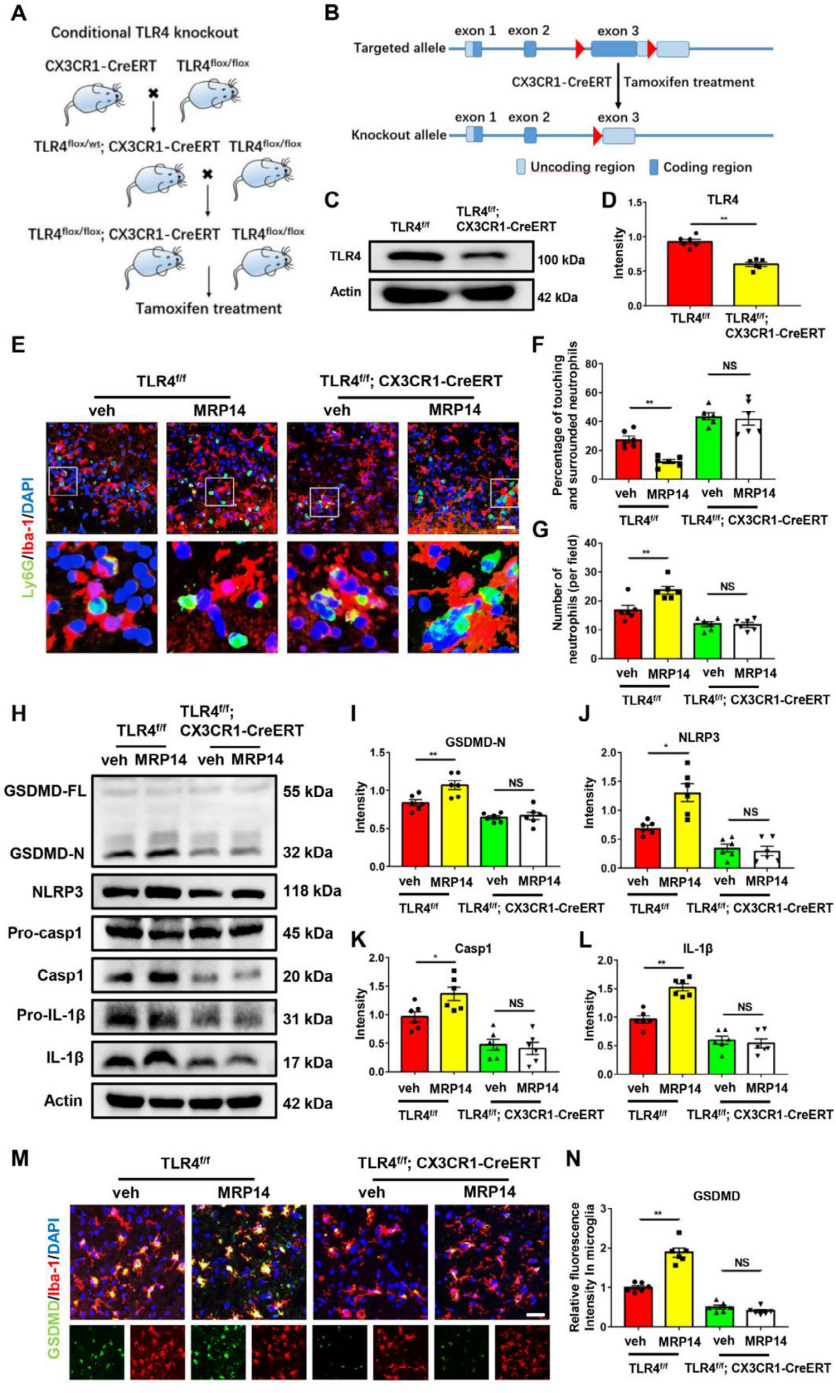
Conditional Knockout of microglial TLR4 alleviates MRP14‐induced impaired phagocytosis and NLRP3 inflammasome‐associated pyroptosis after ischemic stroke. A,B) The schematic diagram showing the breeding strategy for generating mice which were specifically lacking TLR4 expression in microglia (TLR4^flox/flox^; CX3CR1‐CreERT). C,D) Verification of conditionally knockout of TLR4 expression in TLR4^flox/flox^; CX3CR1‐CreERT mice (n = 6/group). E,F) Immunofluorescence staining showing the percentage of neutrophils (green) touching or surrounded by microglia (red) in the ischemic brain of TLR4^flox/flox^ or TLR4^flox/flox^; CX3CR1‐CreERT mice treated with vehicle or recombined MRP14 protein (0.6 mg kg^−1^) 24 h after tMCAO (n = 6/group). G) The neutrophil numbers per high‐power field in the ischemic brain of TLR4^flox/flox^ or TLR4^flox/flox^; CX3CR1‐CreERT mice treated with vehicle or recombined MRP14 protein (0.6 mg kg^−1^) 24 h after tMCAO (n = 6/group). H‐L) Western blot analysis of the expression of GSDMD, NLRP3, pro‐caspase 1, caspase 1, pro‐IL‐1*β* and IL‐1*β* in the ischemic brain of TLR4^flox/flox^ or TLR4^flox/flox^; CX3CR1‐CreERT mice treated with vehicle or recombined MRP14 protein (0.6 mg kg^−1^) 24 h after tMCAO (n = 6/group). M,N) Immunofluorescence staining showing the expression of GSDMD (green) in the microglia (red) in the ischemic brain of TLR4^flox/flox^ or TLR4^flox/flox^; CX3CR1‐CreERT mice treated with vehicle or recombined MRP14 protein (0.6 mg kg^−1^) 24 h after tMCAO (n = 6/group). Data are presented as means ± SEM; unpaired t‐test, one‐way ANOVA. *P < 0.05, **P < 0.01. Scale bar, 50 µm. TLR4, Toll‐like receptor 4; veh, vehicle; TLR4^f/f^, TLR4^flox/flox^; GSDMD, gasdermin D; NLRP3, NOD‐like receptor family, pyrin domain containing 3; IL‐1*β*, interleukin‐1*β*; Casp1, caspase 1.

To validate the involvement of microglial TLR4 in vivo, microglia‐specific TLR4‐deficient mice were generated by breeding TLR4^flox/flox^ mice with CX3CR1‐CreERT mice (**Figure** **6**A,B; Figure S12A,B, Supporting Information). TLR4 expression was effectively reduced in TLR4^flox/flox^; CX3CR1‐CreERT mice (Figure 6C,D). TLR4^flox/flox^; CX3CR1‐CreERT mice, along with their TLR4^flox/flox^ littermates, were subjected to tMCAO followed by the administration of recombinant MRP14 protein.

**Figure 7:**
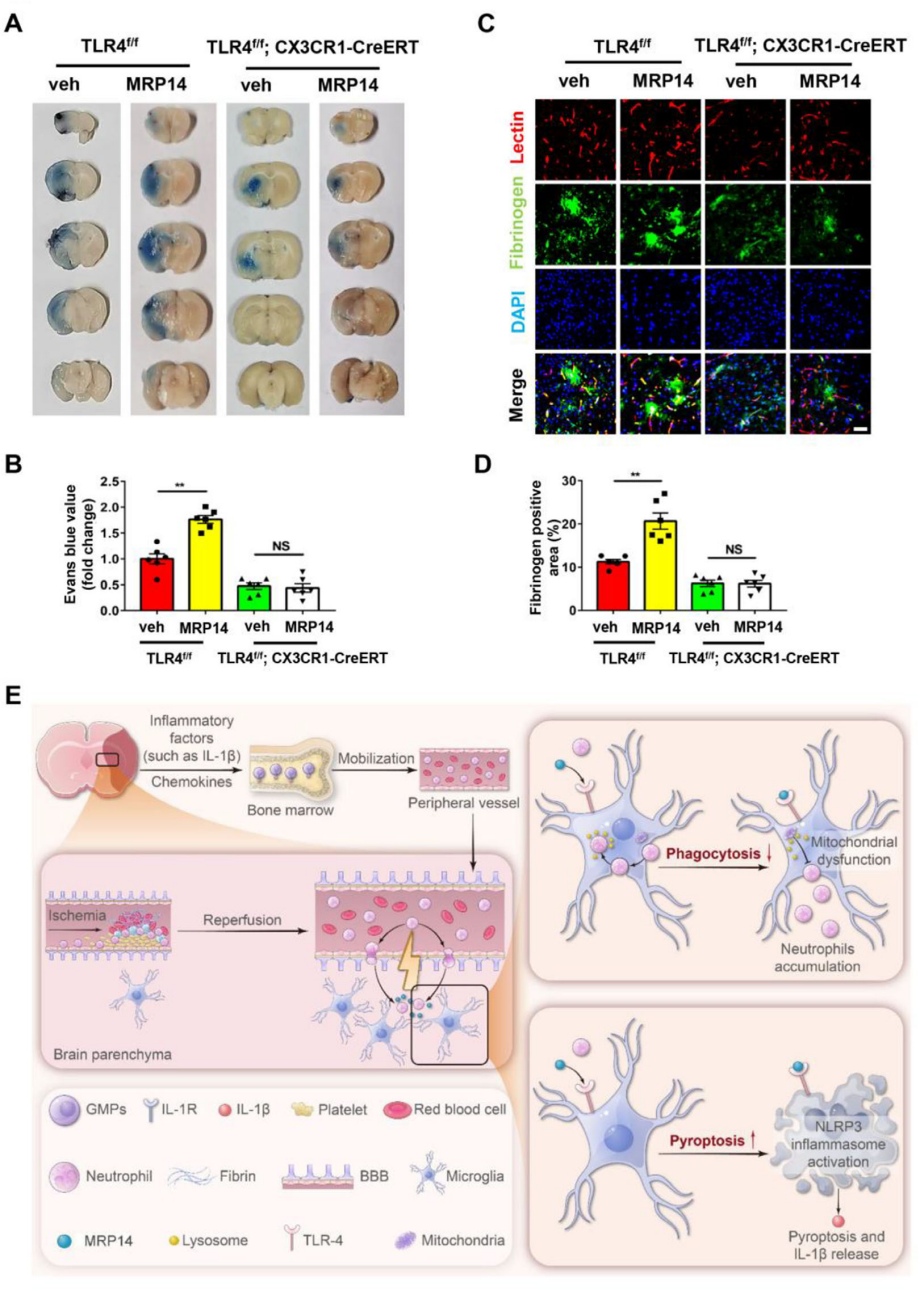
Conditional Knockout of microglial TLR4 alleviates MRP14‐mediated BBB breakdown in the tMCAO mice. A,B) The Evans blue extravasation assay of the TLR4^flox/flox^ or TLR4^flox/flox^; CX3CR1‐CreERT mice treated with vehicle or recombined MRP14 protein (0.6 mg kg^−1^) 24 h after tMCAO (n = 6/group). C,D) Immunofluorescence staining showing the fibrinogen (green) leakage in the ischemic brain of TLR4^flox/flox^ or TLR4^flox/flox^; CX3CR1‐CreERT mice treated with vehicle or recombined MRP14 protein (0.6 mg kg^−1^) 24 h after tMCAO (n = 6/group). E) The schematic illustration showing MRP14‐mediated interactions between neutrophils and microglia after ischemic stroke. Data are presented as means ± SEM; one‐way ANOVA. **P < 0.01. Scale bar, 50 µm. TLR4, Toll‐like receptor 4; BBB, blood brain barrier; tMCAO, transient middle cerebral artery occlusion; mNSS, Modified Neurological Severity Score; TLR4^f/f^, TLR4^flox/flox^; veh, vehicle.

In TLR4^flox/flox^ mice, recombinant MRP14 reduced the percentage of neutrophils touching and surrounded by microglia, meanwhile the overall neutrophil count was increased (Figure 6E‐G). It also upregulated the expression of GSDMD, NLRP3, caspase‐1 and IL‐1*β*, and increased GSDMD fluorescence intensity in microglia (Figure 6H‐N). In contrast, these changes were absent in TLR4^flox/flox^; CX3CR1‐CreERT mice (Figure 6E‐N). Furthermore, recombinant MRP14 protein significantly increased Evans blue and fibrinogen leakage post‐tMCAO in TLR4^flox/flox^ mice, while these effects did not occur in TLR4^flox/flox^; CX3CR1‐CreERT mice (**Figure** **7**A‐D). These results firmly establish that microglial TLR4 is essential for MRP14‐induced impairment of phagocytosis, activation of pyroptosis, and BBB breakdown following cerebral ischemia.

### 2.10 Neutrophil MRP14 Expression can be Modulated by IL‐1*β* via IL‐1R1/MAPK/C/EBP *β* Signaling Pathway

Microglial pyroptosis results in the release of IL‐1*β*, which was reported to regulate the expression of pro‐inflammatory cytokines.^[^ ^19^ ^]^ We found mRNA levels of IL‐1*β* receptor (IL‐1R1) were elevated in peripheral neutrophils from tMCAO mice (Figure S13A, Supporting Information). Recombinant IL‐1*β* significantly increased MRP14 protein levels, and this effect was abolished by the IL‐1R1 inhibitor anakinra (Figure S13B,C, Supporting Information), indicating that IL‐1*β* might also interact with neutrophils to promote MRP14 expression. IL‐1*β* signaling has been shown to activate the NF‐κB and MAPK pathways.^[^ ^19^, ^20^ ^]^ To explore the downstream signaling pathway, we further performed KEGG pathway enrichment analysis of the upregulated DEGs identified in our previous RNA sequencing of peripheral neutrophils from sham and tMCAO mice (Figure S2A, Supporting Information). The analysis showed the activation of the MAPK pathway (Figure S13D, Supporting Information). Western blot analysis confirmed a significant increase in the p‐p38/p38 ratio in neutrophils treated with recombinant IL‐1*β* (Figure S13E,F, Supporting Information). Pre‐treatment with the p38 inhibitor PD 169316 effectively suppressed the IL‐1*β*‐induced elevation of MRP14 in neutrophils (Figure S13G,H, Supporting Information), strongly supporting the role of the p38 MAPK pathway in IL‐1*β*‐induced MRP14 expression in neutrophils.

To identify transcription factors potentially regulating MRP14 expression, bioinformatics analyses were conducted using ALGEEN, GTRD, and AnimalTFDB, with KnockTF employed to predict transcription factors enriched among the DEGs from RNA sequencing. There were five overlapping transcription factors among these four databases: C/EBP *β*, C/EBP α, YY1, STAT6, and PAX5 (Figure S13I, Supporting Information). Previous studies have demonstrated that C/EBP *β* directly promotes MRP14 transcription^[^ ^21^ ^]^ and that its expression is modulated by the MAPK pathway.^[^ ^22^ ^]^ Thus, we performed a dual luciferase assay to assess whether C/EBP *β* could bind to the MRP14 promoter and regulate its expression. The result demonstrated that only the co‐transfection group with MRP14 WT and C/EBP *β* plasmids can lead to an increase in luciferase activity (Figure S13J,K, Supporting Information). Further experiments showed that recombinant IL‐1*β* induced C/EBP *β* expression in neutrophils, an effect significantly inhibited by PD 169316 (Figure S13L,M, Supporting Information). These results conclusively suggest that the IL‐1R1/MAPK/C/EBP *β* signaling pathway contributes to the upregulation of MRP14 expression in neutrophils upon IL‐1*β* treatment.

Previous study has reported that IL‐1*β* could induce neutrophil mobilization from the bone marrow in the model of myocardial infarction.^[^ ^23^ ^]^ Thus, we assumed that IL‐1*β* may similarly promote neutrophil mobilization from the bone marrow after ischemic stroke. Further analysis revealed significantly elevated mRNA levels of the IL‐1R1 in neutrophils isolated from the bone marrow of tMCAO mice, while CXCR4 expression was reduced, and CXCR2 levels remained unchanged (Figure S14A‐C, Supporting Information). These results suggest that IL‐1*β* may facilitate neutrophil mobilization following ischemic stroke.

## 3 Discussion

Neutrophil accumulation in the brain parenchyma is a key driver of neuroinflammation following ischemic stroke. These peripheral neutrophils infiltrate the ischemic brain as early as 15 min after stroke onset, with peak infiltration occurring between 24 and 72 h.^[^ ^4^ ^]^ The balance between neutrophil accumulation and clearance in the ischemic brain critically influences the severity of neuroinflammatory damage. Impaired microglial phagocytosis facilitates neutrophil retention, thereby exacerbating brain injury.^[^ ^11^ ^]^ Our study identified a pivotal role for neutrophil‐derived MRP14 in exacerbating both neutrophil accumulation and neuroinflammation. MRP14 not only serves as a “don't eat me” signal, inhibiting microglial phagocytosis of neutrophils, but also acts as a “kill you” signal, inducing microglial pyroptosis and neuroinflammation. Additionally, IL‐1*β* released from pyroptotic microglia further promotes MRP14 expression in neutrophils and enhances their mobilization from the bone marrow. This cascade of neutrophil accumulation and neuroinflammation ultimately contributes to BBB disruption during ischemic stroke (Figure 7E). These results reveal previously unknown interactions between neutrophils and microglia within the brain parenchyma and support the therapeutic targeting of MRP14 in ischemic stroke.

Despite promising results in pre‐clinical and phase II studies, no direct anti‐inflammatory drugs have demonstrated unequivocal clinical efficacy in phase III trials in stroke treatment.^[^ ^24^ ^]^ The failure of these drugs may be attributed to direct anti‐inflammation treatment act as a double‐edged sword in the context of acute ischemic stroke, which not only alleviates inflammatory damage and improves neurological function, but also compromises the body's immune defense, potentially leading to life‐threatening infections.^[^ ^24^ ^]^ Moreover, specific subsets of inflammatory cells, such as N2 and CD177⁺ neutrophils,^[^ ^4^, ^25^ ^]^ may exert protective effects following ischemic stroke. Therefore, direct anti‐inflammatory therapies may inhibit the protective effects of these cells. Ultimately, these adverse effects may counteract their benefits and influence patient prognosis. In the current study, we found that selective MRP14 targeting effectively inhibits the inflammatory cascade within ischemic brain tissue while minimizing the adverse effects commonly associated with direct anti‐inflammatory treatments.

Our study identified neutrophils as the primary source of MRP14 at 24 h after tMCAO. However, a recently published study demonstrated a role for microglia/macrophage‐derived MRP14 between 3 and 21 days after tMCAO.^[^ ^26^ ^]^ Notwithstanding, their findings were not inconsistent with ours. First, it is reported that MRP14 is predominantly expressed in neutrophils and monocytes in peripheral blood, with only limited expression in brain parenchymal microglia.^[^ ^27^ ^]^ In peripheral blood, MRP14 constitutes ≈45% of the total cytosolic protein in neutrophils, while monocytes express ≈40‐fold lower levels.^[^ ^28^ ^]^ Second, neutrophils reach their peak infiltration 1–2 days post‐ischemia, corresponding with the increase in brain MRP14 expression observed in our study. In contrast, monocyte infiltration begins one day post‐ischemia, peaking at 4th day,^[^ ^29^ ^]^ and occurs at levels significantly lower than those of neutrophils.^[^ ^30^ ^]^ Immunofluorescence staining in this study further confirmed that MRP14 predominantly co‐localizes with neutrophils, with minimal expression in microglia within ischemic brain tissue. Finally, bone marrow neutrophil transfer into MRP14 KO mice exacerbated BBB damage at 24 h after tMCAO, underscoring the role of neutrophil‐derived MRP14 in ischemia‐reperfusion injury. These results strongly demonstrate that neutrophils are the primary source of elevated MRP14 during the acute stage of ischemic stroke.

Recent studies have shown that reactive microglia can phagocytose and clear infiltrating peripheral neutrophils recruited to the ischemic brain, thus playing a pivotal role in protecting against neutrophil‐induced tissue damage after stroke.^[^ ^11^ ^]^ Our findings suggest that MRP14 acts as a “don't eat me” signal, protecting neutrophils from microglial phagocytosis post‐stroke. Compared with WT mice, MRP14 KO mice exhibited significantly enhanced microglial phagocytosis after tMCAO. In vitro experiments further confirmed the inhibitory effects of MRP14 on microglial phagocytosis of neutrophils. Additionally, MRP14 induces mitochondrial dysfunction in microglia, which likely explains impaired phagocytic capacity following MRP14 exposure. This observation is consistent with previous studies showing that elevated MRP14 levels can cause mitochondrial dysfunction in models of acute myocardial infarction and sepsis.^[^ ^17^, ^31^ ^]^

In addition, MRP14 also functions as a “kill you” signal, promoting microglial pyroptosis and the subsequent release of inflammatory mediators, such as IL‐1*β*, which exacerbates BBB damage following AIS. Both pharmacological inhibition of MRP14 using paquinimod and genetic KO of MRP14 significantly attenuated NLRP3 inflammasome‐associated pyroptosis and mitigated BBB disruption after tMCAO. Furthermore, IL‐1*β* has been reported to enhance neutrophil mobilization from the bone marrow in conditions like myocardial infarction.^[^ ^23^ ^]^ Administration of the IL‐1R1 antagonist anakinra similarly reduced leukocyte counts, particularly neutrophils, in patients with stroke.^[^ ^32^ ^]^ Our findings support these observations by showing significantly increased IL‐1R1 expression in bone marrow neutrophils post‐tMCAO, reinforcing the role of IL‐1*β* in promoting neutrophil mobilization. Moreover, IL‐1*β* induces MRP14 expression in neutrophils via the IL‐1R1/MAPK/C/EBP *β* signaling pathway, establishing a harmful feedback loop that amplifies MRP14‐induced damage.

Previous studies have identified TLR4 and RAGE as receptors for extracellular MRP14.^[^ ^27^ ^]^ In our study, receptor‐blocking experiments demonstrated that MRP14‐induced microglial responses were dependent on TLR4 rather than RAGE. Using microglia‐specific TLR4‐deficient mice in the tMCAO model, it was confirmed that MRP14 impairs microglial phagocytosis and promotes pyroptosis through TLR4. As a member of the pattern recognition receptor family, TLR4 plays a critical role in immune responses and phagocytic function.^[^ ^33^ ^]^ Numerous studies have shown that TLR4 deficiency exerts neuroprotective effects by reducing post‐stroke inflammation.^[^ ^34^, ^35^ ^]^ Additionally, our findings indicated that MRP14 triggers mitochondrial dysfunction through TLR4, thereby reducing the energy supply needed for microglial phagocytosis of neutrophils following tMCAO. This mechanism aligns with previous studies linking MRP14 to mitochondrial impairment in myocardial ischemia‐reperfusion injury and sepsis.^[^ ^17^, ^31^ ^]^

This study has several limitations that warrant consideration. First, the precise mechanism by which ischemic stroke induces the release of MRP14 from neutrophils remains unclear. Second, whether paquinimod‐mediated inhibition of MRP14 can prevent AIS‐induced BBB damage in clinical settings remains unknown. Third, the potential adverse effects associated with MRP14 inhibition were not fully explored. Because intracellular MRP14 is involved in cytoskeletal regulation and arachidonic acid metabolism.^[^ ^27^ ^]^ Therefore, selectively targeting extracellular MRP14, possibly through antibodies or nanoparticles, could represent a novel adjunctive therapy for AIS, minimizing interference with essential intracellular processes.

## 4 Experimental Section

### Patient Sample

Patients for this study were drawn from the TRAIS cohort, which retrospectively compiled data from all consecutive patients with AIS admitted to 14 stroke centers in China (ChiCTR2000033456). All participants provided informed consent, and the study adhered to the ethical standards outlined in the Declaration of Helsinki. The local institutional review boards approved all study protocols (Wuhan, China, 2020IEC‐0273).

Plasma samples were collected from patients with AIS within 24 h of stroke onset. Edema was assessed using the SITS‐MOST grading system, where signs of focal brain edema typically involve the narrowing of cerebrospinal fluid spaces, such as the effacement of cortical sulci or ventricular compression.^[^ ^36^, ^37^ ^]^ Based on MRI or CT scans conducted within 24–72 h post‐stroke, patients were categorized as having no cerebral edema (cerebral edema‐0), cerebral edema affecting less than one‐third of the hemisphere (cerebral edema‐1), cerebral edema involving more than one‐third of the hemisphere (cerebral edema‐2), or cerebral edema resulting in midline shift (cerebral edema‐3). For the purposes of this study, patients classified as cerebral edema‐0 and ‐1 were grouped as having mild cerebral edema, while those with cerebral edema‐2 and ‐3 were grouped as having severe cerebral edema.

### Animals

C57BL/6J mice (8 weeks old) were obtained from Hubei Biont Biological Technology Co., Ltd. (Wuhan, China), and C57BL/6J‐*MRP14^em1C^*/Cya (MRP14 KO, S‐KO‐16221) mice were sourced from Cyagen Biosciences Inc. (Suzhou, China). Additionally, B6.129P2(C)‐*Cx3cr1^2.1 (cre/ERT2) Jung^*/J (CX3CR1‐CreERT, #020940) mice were purchased from Jackson Laboratory (Bar Harbor, USA), while C57BL/6‐*Tlr4^em1 (flox) Smoc^* (TLR4^flox/flox^, NM‐CKO‐200230) mice were acquired from Shanghai Model Organisms Center, Inc. (Shanghai, China). To generate microglia‐specific TLR4‐deficient mice, TLR4^flox/flox^ mice were crossed with CX3CR1‐CreERT mice. Six‐week‐old male TLR4^flox/flox^; CX3CR1‐CreERT mice and littermate TLR4^flox/flox^ mice underwent tamoxifen (Beyotime Biotechnology, China) treatment to induce Cre recombinase expression (intraperitoneal injection, 75 mg kg^−1^ for 5 consecutive days). All animals were housed under a 12‐h light‐dark cycle at controlled temperatures (20–25 °C) and humidity levels (40–60%) with ad libitum access to food and water. The medical ethics committee of Tongji Medical College, Huazhong University of Science and Technology, approved all animal protocols (Wuhan, China, 2023IACUC‐4223), which were conducted in compliance with the Guide for the Care and Use of Laboratory Animals.

### Establishment of the tMCAO Model

The tMCAO model was performed on adult mice (22–25 g) as previously described.^[^ ^38^ ^]^ In brief, mice were anesthetized using isoflurane inhalation and a midline incision was made to fully expose the right common carotid arteries (CCA), internal carotid arteries (ICA) and external carotid arteries (ECA). Then, a 6‐0 silicon‐coated nylon suture (Yushun Biotech, China) was inserted into the ECA, advanced through the CCA, and finally reached the origin of the middle cerebral artery in the ICA to block the blood flow. One hour after ischemia, the suture was removed to achieve reperfusion to the ischemic brain. The sham group was exposed to the same procedures aside from middle cerebral artery occlusion. The temperature was controlled throughout the surgery and the cerebral blood flow was measured to ascertain ischemia and reperfusion. MRP14 inhibitor paquinimod (10 mg kg^−1^, MedChemExpress, USA), or recombined MRP14 protein (0.6 mg kg^−1^, Cloud‐Clone Corp, China) was intravenously injected into the tMCAO mice immediately after the removal of suture as previously described.^[^ ^39^ ^]^ The normal saline was administrated at the same volume as vehicle control.

### Neutrophil Depletion

To deplete neutrophils, anti–mouse Ly6G antibody (BioXCell, USA) were intraperitoneally injected into mice at a dose of 10 mg kg^−1^ for consecutive two days before tMCAO procedure. Control mice were administrated with the same dose of IgG.

### OGD/R

OGD/R was conducted as previously described.^[^ ^38^ ^]^ Briefly, the culture medium was replaced with Dulbecco's Modified Eagle Medium (DMEM) without glucose. Then, the cells were transferred into the anaerobic incubator (95% N_2_ and 5% CO_2_) at 37 °C. After 6 h of OGD, the cells were cultured with normal medium in the normoxic conditional incubator for 24 h at 37 °C to allow reperfusion.

### Neutrophil Isolation and Transfer

The bone marrow neutrophils were sorted from the mouse femurs and tibias and peripheral neutrophils were sorted from the mouse peripheral blood using Histopaque gradients (Sigma–Aldrich, USA).^[^ ^40^ ^]^ In brief, 3 mL Histopaque 1119 was added into the 15 mL tube, and then 3 mL Histopaque 1077 was gently layered on the top of Histopaque 1119, followed by pipetting 1 mL cells suspensions. Then, the tube was centrifuged at 872 × g for 30 min at room temperature without brake. The cell ring at the interface of the Histopaque 1077 and Histopaque 1119 layers was the neutrophils. After washed 3 times with phosphate buffer solution (PBS, Servicebio, China), neutrophils were suspended with RPMI (Gibco BRL, USA) containing 10% fetal bovine serum (FBS, Gibco BRL, USA) for the subsequent experiments. For tracing the isolated bone marrow neutrophils, MIRB (Biophysics Assay Laboratory, Inc. USA) was added into the culture at 12.5 µg mL^−1^ concentration for 24 h. Then, neutrophils were harvested and injected into recipient mouse via tail vein (5 × 10^6^ neutrophils per mouse). Peripheral neutrophils were treated with recombined IL‐1*β* (MedChemExpress, USA) at a concentration of 10 ng mL^−1^ for 4 h, and anakinra (10 µg mL^−1^, MedChemExpress, USA) or PD 169316 (10 µM, MedChemExpress, USA) was added 30 min before IL‐1*β* treatment.

### Primary Microglia and Astrocyte Cultures

Primary microglia and astrocyte cultures were isolated from newborn mice pups (1–3 days old) as described previously.^[^ ^41^ ^]^ The cortices were dissected from the pups’ brains and the meninges were removed with microscope forceps. Then, the brain tissues were digested in 0.25% trypsin/EDTA solution for 15 min at 37 °C and prepared for cell suspension to seed onto the poly‐d‐lysine‐coated culture flasks in the medium containing 10% FBS and 1% penicillin/streptomycin. The cultures were maintained for 14 days and the medium was replaced every 3 days to generate mixed glial system. In order to separate primary microglia from astrocytes, the culture flasks were placed on a gyratory shaker (250 rpm for 1 h) and then the detached microglia were collected and seeded on new culture plates for further studies. The remaining cells were used as primary astrocytes. The primary microglia were stimulated with recombined MRP14 (Abcam, China) protein at a dose of 1 µg mL^−1^ for 24 h, followed by ATP (Sigma–Aldrich, USA) treatment for 30 min. The LPS (Sigma–Aldrich, USA) was added at 500 ng mL^−1^ 6 h before recombined MRP14 or ATP treatment. Resatorvid (the TLR4 inhibitor, MedChemExpress, USA), FPS‐ZM1 (the RAGE inhibitor, MedChemExpress, USA) and JSH‐23 (the NF‐κB inhibitor, MedChemExpress, USA) were added into the microglial culture at 100 nM, 10 µM and 10 µM, respectively.

### Phagocytosis Assay In Vitro

First, primary microglia were subjected to OGD stimulus for 6 h and the recombined MRP14 protein was added at the beginning of reperfusion for another 24 h. At the same time, neutrophils were isolated from tMCAO mice. Then, primary microglia were co‐cultured with neutrophils for 12 h (microglia: neutrophils, 1: 3) and washed with PBS to clear suspended neutrophils. The phagocytized neutrophils by microglia were measured using immunofluorescence staining and Western blot analysis.

### ATP Measurements

The intracellular ATP level was detected using the ATP Assay Kit (Beyotime Biotechnology, China). In brief, microglia were collected and incubated with 200 µL lysis buffer, followed by centrifugation at 12000 x g for 5 min. Then, 20 µL supernatant was added into the 96 cell wells which contains 100 µL of the ATP reaction buffer. The luminescence of each cell well was determined by a microplate reader.

### Measurement of Mitochondrial Membrane Potential

The changes in mitochondrial membrane potential were detected using the enhanced mitochondrial membrane potential assay kit with JC‐1 (Beyotime Biotechnology, China). The treated microglia were collected and incubated with 1 mL of JC‐1 work solution for 20 min at 37 °C. Then, microglia were washed with staining buffer and analyzed on flow cytometry. The fluorescence change from red to green suggests the breakdown of mitochondrial membrane potential.

### Single‐Cell Library Preparation and Sequencing

Single‐cell suspensions were prepared as described previously.^[^ ^42^ ^]^ Briefly, the ischemic brain was harvested from WT and MRP14 KO mice 24 h after the tMCAO procedure, followed by preparation using the adult brain dissociation kit (Miltenyi Biotec, Germany). Cell suspensions with viability exceeding 85% were used for subsequent library preparation. The cells were then loaded onto a Chip A Single Cell Kit v2.0 (MobiDrop, cat. no. S050100201), and droplets were generated with a MobiNova‐100 (MobiDrop, cat. no. A1A40001). Each droplet encapsulated a single cell along with a gel bead coated with millions of oligonucleotides containing unique cell barcodes. The encapsulated droplets were photo‐cleaved using a MobiNovaSP‐100 (MobiDrop, cat. no. A2A40001), allowing mRNA to bind to the oligonucleotide‐coated gel beads. After reverse transcription, the cDNAs were amplified to construct the library using the 3′ Single Index Kit (MobiDrop (Zhejiang) Co., Ltd., cat. no. S050300201) and the High Throughput Single Cell 3′ RNA‐Seq Kit v2.0 (MobiDrop (Zhejiang) Co., Ltd., cat. no. S050200201), and the libraries were sequenced on the Illumina NovaSeq 6000 System.

### Fundamental Analysis of scRNA‐seq Data

Basic cell clustering analysis was performed using the Seurat 4.3.0 R package. Cells with gene numbers less than 200 or in the top 1%, or with mitochondrial gene content exceeding 25% were considered aberrant and excluded from further analysis. Dimensionality reduction was performed using principal component analysis (PCA), followed by identification of different clusters using the FindClusters function, which were visualized using UMAP.

### Differentially Expressed Gene and Enrichment Analysis

To identify DEGs in each cluster, the FindAllMarkers or FindMarkers function was employed. The top 20 marker genes from each cluster were subjected to GO, KEGG, and Reactome pathway enrichment analysis using KOBAS software with Benjamini‐Hochberg correction, and the results were visualized using the R package.

### Calculation of Gene Set Module Score

The AddModuleScore method was used to calculate the average expression levels of selected gene sets across clusters, with the results visualized as violin plots in Seurat (4.2.0). The MSigDB molecular signature database (https://www.gsea‐msigdb.org/gsea/msigdb/index.jsp) was used to select gene sets related to each cell death pattern, as listed in Table S2 (Supporting Information).

### RNA Sequencing of Neutrophils

Neutrophils were isolated from the peripheral blood of AIS patients with mild and severe cerebral edema, and sham and tMCAO mice. Total RNA was extracted using TRIzol reagent (Enzyme, China) according to the manufacturer's protocol and subsequently sent to Seqhealth Genomics (China) for RNA sequencing. In summary, mRNA was purified from total RNA and fragmented into smaller pieces, which were then used for cDNA library construction. Sequencing was conducted on the Illumina Hiseq platform, with ten replicates performed. DEGs were identified using DESeq2 (v1.4.5), with genes displaying a fold change greater than 1 and a *p*‐value of less than 0.05 considered significant. REACTOME pathway analysis was utilized to elucidate the altered intracellular signaling pathways in the various groups.

### Flow Cytometry

Flow cytometric analysis of the expression of MRP14 in the blood neutrophils and monocytes was conducted as previously described.^[^ ^43^ ^]^ In brief, the following fluorescence‐conjugated antibodies were used to stain the single cell suspensions: APC Mouse Anti‐Human CD14 (M5E2, BD Pharmingen), PE‐Cy7 Mouse Anti‐Human CD16 (3G8, BD Pharmingen), BV421 Mouse Anti‐Human CD11b (ICRF44, BD Pharmingen), FITC Mouse Anti‐Human CD15 (HI98, BD Pharmingen), and PE Conjugated MRP14 Rabbit mAb (D5O6O, Cell Signaling Technology). Then, the cells were analyzed with the flow cytometer (BD Pharmingen, USA) and FlowJo software (Treestar, USA).

### Evans Blue Dye

Evans blue dye (4%, 4 mL kg^−1^, Sigma–Aldrich, USA) was injected into the mice via the tail vein. Four hours after circulation, the mice were sacrificed and transcardially perfused with PBS. Then, the mice brains were sliced and homogenized in 50% trichloroacetic acid (Sigma–Aldrich, USA) at 65 °C for 24 h. After centrifuged at 12, 000 × g for 20 min, the supernatant was collected and measured by spectrophotometry at absorbance of 620 nm to quantify the leakage of Evans blue dye.

### Measurements of BBB Permeability

To determine the permeability of the in vitro BBB model, the TEER value and FITC‐dextran trans‐endothelial permeability were measured. The endothelial voltohmmeter (EVOM, World Precision Instruments, USA) was used to calculate the TEER value following the manufacturer's instruction: TEER value (Ω × cm^2^) = [(resistance of cells insert – resistance of blank insert) × superficial area of the insert]. For the FITC‐dextran trans‐endothelial permeability assay, 1 mg mL^−1^ of FITC‐dextran (MW, 70, 000 kd, Sigma–Aldrich, USA) was first added into the upper chamber for 2 h and then the absorbance in the lower chamber was measured via a microplate reader at 450 nm.

### Dual‐Luciferase Assay

The MRP14 luciferase reporter plasmid, Renilla control reporter plasmid and C/EBP *β* plasmid were co‐transfected into the HEK293T cells. After 48 h of transfection, HEK293T cells were lysed and the firefly and Renilla luciferase activity were measured using the Dual Luciferase Reporter Assay Kit (Vazyme, China) following the manufacturer's protocol. The Renilla control reporter was used as an internal control.

### Western Blot Analysis

Cell and tissue samples were lysed with the RIPA buffer (Beyotime Biotechnology, China) which contains 1% protease inhibitors (Beyotime Biotechnology, China) to extract proteins. Then, an equal amount of protein extract was added into the SDS‐PAGE gels and transferred onto the PVDF membranes (Millipore, USA). 5% skim milk buffer was used to block nonspecific binding and the transferred PVDF membranes was incubated with primary antibodies overnight at 4 °C: MRP14 (1:1000, 73425, Cell Signaling Technology), Occludin (1:1000, 13409‐1‐AP, Proteintech), ZO‐1 (1:1000, 21773‐1‐AP, Proteintech), VE‐Cadherin (1:1000, AF1002, RD system), MPO (1:1000, 22225‐1‐AP, Proteintech), Caspase‐1 (1:50, A0964, Abclonal), IL‐1*β* (1:500, A16288, Abclonal), NLRP3 (1:500, T55651, Abmart), GSDMD (1:1000, ab209845, Abcam), p65 (1:500, A19653, Abclonal), p‐p65 (1:500, TA2006, Abmart), IκB (1:500, A1187, Abclonal), p‐IκB (1:500, AP0707, Abclonal), Tubulin (1:1000, M20005, Abmart), p‐p38 (1:1000, 4511, Cell Signaling Technology), p38 (1:1000, 8690, Cell Signaling Technology), C/EBP *β* (1:1000, 23431‐1‐AP, Proteintech), TLR4 (1:1000, 66350‐1‐Ig, Proteintech), *β*‐actin (1:1000, AC026, Abclonal), H3 (1:1000, 17168‐1‐AP, Proteintech). The membranes were then incubated with secondary antibodies for 2 h at room temperature and visualized using ECL solution on the chemiluminescence system (UVP, USA). Image J software was used to detect the intensity of the bands.

### Immunofluorescence

The brain sections were first heat‐treated with citrate buffer solution to retrieve the antigen and then incubated with 0.3% Triton X‐100 and 10% donkey serum for 1 h at room temperature. Next, the sections were incubated with the following primary antibodies overnight at 4 °C: MRP14 (1:50, 73425, Cell Signaling Technology), Ly6G (1:50, ab25377, Abcam), VE‐Cadherin (1:50, AF1002, RD system), Fibrinogen (1:50, ab118533, Abcam), Lectin (1:50, DL‐1177‐1, Vector laboratories), GSDMD (1:50, ab209845, Abcam), Iba‐1 (1:50, ab5076, Abcam), CD68 (1:50, GB113109‐100, Servicebio). After washed with PBST buffer, fluorescein‐conjugated secondary antibodies were added. All brain sections were captured using the confocal microscope (Nikon, Japan).

### Enzyme‐Linked Immunosorbent Assay (ELISA)

After centrifuged at 400 × g for 10 min, the supernatant of cell culture medium and plasma samples were collected and stored at −80 °C for further measurement. The MRP14 and PGLYRP1 levels in the plasma of patients and mice, and the IL‐1*β* levels in the cell culture supernatant were quantified using the ELISA kits (Bioswamp, China) following the manufacturer's instructions.

### Real‐Time Quantitative PCR

The RNA extraction reagent (Vazyme, China) was used to extract total RNAs from neutrophils and brain tissues. Then, total RNA was reverse‐transcribed into cDNAs by HiScript III‐RT SuperMix (Vazyme, China) and amplified using SYBR Green Mix (Vazyme, China) according to the manuals. Primers for PCR were listed in Table S3 (Supporting Information). The comparative 2‐ΔΔCt method against *β*‐actin was applied to quantify the gene expression.

### Statistical Analysis

GraphPad Prism 9.0 software was used to perform the statistical analyses presented in this study. All data were presented as mean ± SEM and the sample size (n) is indicated in the figure legends. The Shapiro‐Wilk test was used to assess normality. For normally distributed data, an unpaired two‐tailed Student's t‐test was applied for comparisons between two groups, while one‐way ANOVA followed by Tukey's post hoc test was used for comparisons involving more than two groups. For data not following a normal distribution, the Mann‐Whitney test was employed for two‐group comparisons, and Kruskal‐Wallis test followed by Dunn's test was used for nonparametric analysis involving more than two groups.

## Conflict of Interest

The authors declare no conflict of interest.

## Supporting information



Supporting Information

## Data Availability

The data that support the findings of this study are available from the corresponding author upon reasonable request.
