# Supplementary material for: Neutrophil Mobilization Triggers Microglial Functional Change to Exacerbate Cerebral Ischemia‐Reperfusion Injury
Source: Adv Sci (Weinh). 2025 Jun 25;12(36):e03722. doi: 10.1002/advs.202503722 (PMC12462934; doi:10.1002/advs.202503722)
Supplement: Supplementary file 1 — Supporting Information [file ADVS-12-e03722-s001.docx]

**Supporting Information**

**Neutrophil Mobilization Triggers Microglial Functional Change to Exacerbate Cerebral Ischemia-Reperfusion Injury**

*Huijuan Jin, Zhifang Li, Senwei Tan, Qinghui Xiao, Qingcan Li, Jiao Ye, Yifan Zhou, Yan Wan, Qiang Liu, Bijoy K Menon, Bo Hu**

H. Jin, Z. Li, S. Tan, Q. Xiao, Q. Li, J. Ye, Y. Zhou, Y. Wan, B. Hu

Department of Neurology

Union Hospital

Tongji Medical College

Huazhong University of Science and Technology

Wuhan 430022, China.

E-mail: hubo@mail.hust.edu.cn

Q. Liu

Department of Neurology

Tianjin Neurological Institute

Tianjin Medical University General Hospital

Tianjin 300052, China.

B.-K. Menon

Department of Community Health Sciences, Department of Clinical Neurosciences, Department of Radiology, and Hotchkiss Brain Institute

Cumming School of Medicine

University of Calgary

AB T2N 1N4, Canada.

H. Jin and Z. Li contributed equally to this article.

Funding: This work was supported by the National Natural Science Foundation of China (Grants: 82090044, 82330040, and 81820108010 to Bo Hu; 82171306 and 82471343 to Huijuan Jin; 81901214 to Yan Wan), the National Key Research and Development Program of China (2024YFC3044800 to Bo Hu), and the Hubei Province Key R&D Program (No. 2022BCA008 to Bo Hu).

**Table S1-3**

**Figure S1-14**

**Supplemental Table and Legends**

**Table S1.** Clinical characteristics of patients with AIS exhibiting different cerebral edema grades enrolled from the TRAIS cohort.

|  | Total (n = 414) | 0 (n = 202) | 1 (n = 132) | 2 (n = 38) | 3 (n = 42) | Statistic | *p v*alue |
| --- | --- | --- | --- | --- | --- | --- | --- |
|  |  |  |  |  |  |  |  |
| **Demographic characteristics** | | | | | | | |
| Age (years), Mean ± SD | 66.64 ±  11.77 | 66.23 ± 11.83 | 66.66 ± 11.27 | 67.55 ± 11.63 | 67.69 ± 13.35 | F=0.27 | 0.848 |
| Male, n (%) | 277 (66.91) | 136 (67.33) | 90 (68.18) | 29 (76.32) | 22 (52.38) | χ²=5.63 | 0.131 |
| **Medical history** | | | | | | | |
| Smoking, n (%) | 143 (34.54) | 74 (36.63) | 43 (32.58) | 14 (36.84) | 12 (28.57) | χ²=1.37 | 0.713 |
| Alcohol, n (%) | 98 (23.67) | 48 (23.76) | 31 (23.48) | 8 (21.05) | 11 (26.19) | χ²=0.30 | 0.961 |
| Ischemic stroke, n (%) | 57 (13.77) | 22 (10.89) | 22 (16.67) | 7 (18.42) | 6 (14.29) | χ²=3.04 | 0.385 |
| Intracerebral hemorrhage, n (%) | 11 (2.66) | 4 (1.98) | 3 (2.27) | 2 (5.26) | 2 (4.76) | - | 0.355 |
| Hypertension, n (%) | 271 (65.46) | 129 (63.86) | 87 (65.91) | 27 (71.05) | 28 (66.67) | χ²=0.79 | 0.851 |
| Diabetes mellitus, n (%) | 107 (25.85) | 60 (29.70) | 33 (25.00) | 9 (23.68) | 5 (11.90) | χ²=5.97 | 0.113 |
| Hyperlipemia, n (%) | 64 (15.46) | 39 (19.31) | 13 (9.85) | 5 (13.16) | 7 (16.67) | χ²=5.67 | 0.129 |
| Coronary Heart Disease, n (%) | 39 (9.42) | 14 (6.93) | 13 (9.85) | 6 (15.79) | 6 (14.29) | - | 0.173 |
| **Admission characteristics** | | | | | | | |
| NIHSS, M (Q₁, Q₃) | 4(2, 9) | 2 (1,4) | 4.5 (2,8) | 10.5 (5,14) | 13 (10,18) | χ²=120.42 | <.001 |

AIS, acute ischemic stroke; SD: standard deviation; M: Median; Q₁: 1st Quartile; Q₃: 3st Quartile.

**Table S2.** Gene sets related to cell death patterns.

| Apoptosis | Pyroptosis | Ferroptosis | Necroptosis |
| --- | --- | --- | --- |
| AATF | BAK1 | ABCC1 | TNF |
| ABL1 | BAX | ACACA | TNFRSF1A |
| ACAA2 | CASP1 | ACO1 | TRADD |
| ACKR3 | CASP3 | ACSF2 | TRAF2 |
| ACVR1 | CASP4 | ACSL1 | TRAF5 |
| ACVR1B | CASP5 | ACSL3 | RIPK1 |
| ADORA1 | CASP6 | ACSL4 | BIRC2 |
| AEN | CASP8 | ACSL5 | BIRC3 |
| AGT | CASP9 | ACSL6 | XIAP |
| AGTR2 | CHMP2A | AIFM2 | RBCK1 |
| AIFM1 | CHMP2B | AKR1C1 | RNF31 |
| AKT1 | CHMP3 | AKR1C2 | SHARPIN |
| ANXA6 | CHMP4A | AKR1C3 | SPATA2L |
| APAF1 | CHMP4B | ALOX12 | SPATA2 |
| APPL1 | CHMP4C | ALOX15 | CYLD |
| AR | CHMP6 | ALOX5 | FADD |
| ARHGEF2 | CHMP7 | ATG5 | CASP8 |
| ARL6IP5 | CYCS | ATG7 | CFLAR |
| ARMC10 | ELANE | ATP5MC3 | RIPK3 |
| ARRB2 | GPX4 | BACH1 | CYBB |
| ASAH2 | GSDMB | CARS | CAMK2A |
| ATF3 | GSDMC | CBS | CAMK2D |
| ATF4 | GSDMD | CD44 | CAMK2B |
| ATM | GSDME | CHAC1 | CAMK2G |
| ATP2A1 | GZMB | CISD1 | SLC25A4 |
| ATP2A3 | HMGB1 | CP | SLC25A5 |
| ATP5IF1 | IL18 | CRYAB | SLC25A6 |
| AVP | IL1A | CS | SLC25A31 |
| BAD | IL1B | CYBB | PPID |
| BAG3 | IRF1 | DPP4 | VDAC1 |
| BAG5 | IRF2 | EMC2 | VDAC2 |
| BAG6 | NLRC4 | FADS2 | VDAC3 |
| BAK1 | NLRP1 | FANCD2 | GLUD2 |
| BAX | NLRP2 | FDFT1 | GLUD1 |
| BBC3 | NLRP3 | FTH1 | GLUL |
| BCAP31 | NLRP6 | FTL | PYGL |
| BCL10 | NLRP7 | FTMT | PYGM |
| BCL2 | NOD1 | G6PD | PYGB |
| BCL2A1 | PLCG1 | GCLC | MAPK8 |
| BCL2L1 | PJVK | GCLM | MAPK10 |
| BCL2L10 | PRKACA | GLS2 | MAPK9 |
| BCL2L11 | PYCARD | GOT1 | FTH1 |
| BCL2L12 | SCAF11 | GPX4 | FTL |
| BCL2L14 | TINAP | GSS | PLA2G4E |
| BCL2L2 | TNF | HMGCR | PLA2G4A |
| BCL3 | TP53 | HMOX1 | JMJD7-PLA2G4B |
| BCLAF1 | TP63 | HSBP1 | PLA2G4B |
| BDKRB2 | AIM2 | HSPB1 | PLA2G4C |
| BDNF | GSDMA | IREB2 | PLA2G4D |
| BECN1 | IL6 | KEAP1 | PLA2G4F |
| BID | NOD2 | LPCAT3 | ALOX15 |
| BIK | TIRAP | MAP1LC3A | CAPN1 |
| BIRC6 |  | MAP1LC3B | CAPN2 |
| BLOC1S2 |  | MAP1LC3C | SMPD1 |
| BMF |  | MT1G | MLKL |
| BMP4 |  | NCOA4 | PGAM5 |
| BMP5 |  | NFE2L2 | DNM1L |
| BMPR1B |  | NFS1 | NLRP3 |
| BNIP3 |  | NOX1 | PYCARD |
| BNIP3L |  | NQO1 | CASP1 |
| BOK |  | NRF2 | IL1B |
| BRCA1 |  | OTUB1 | CHMP2A |
| BRCA2 |  | PCBP1 | CHMP2B |
| BRSK2 |  | PCBP2 | CHMP3 |
| BTK |  | PEBP1 | RNF103-CHMP3 |
| CAAP1 |  | PGD | CHMP4B |
| CASP1 |  | PHKG2 | CHMP4A |
| CASP10 |  | PRNP | CHMP4C |
| CASP12 |  | PROM2 | CHMP6 |
| CASP2 |  | PTGS2 | VPS4B |
| CASP3 |  | RPL8 | VPS4A |
| CASP4 |  | SAT1 | CHMP1B |
| CASP5 |  | SAT2 | CHMP1A |
| CASP8 |  | SLC11A2 | CHMP5 |
| CASP8AP2 | | SLC1A5 | CHMP7 |
| CASP9 |  | SLC39A14 | TRPM7 |
| CAV1 |  | SLC39A8 | IL1A |
| CCAR2 |  | SLC3A2 | IL33 |
| CCK |  | SLC40A1 | HMGB1 |
| CD14 |  | SLC7A11 | TNFSF10 |
| CD24 |  | SQLE | TNFRSF10A |
| CD27 |  | STEAP3 | TNFRSF10B |
| CD28 |  | TF | FASLG |
| CD38 |  | TFRC | FAS |
| CD3E |  | TP53 | FAF1 |
| CD44 |  | VDAC2 | IFNA1 |
| CD5 |  | VDAC3 | IFNA2 |
| CD70 |  | ZEB1 | IFNA4 |
| CD74 |  |  | IFNA5 |
| CDIP1 |  |  | IFNA6 |
| CDKN1A |  |  | IFNA7 |
| CDKN2D |  |  | IFNA8 |
| CEBPB |  |  | IFNA10 |
| CFLAR |  |  | IFNA13 |
| CHAC1 |  |  | IFNA14 |
| CHCHD10 |  |  | IFNA16 |
| CHEK2 |  |  | IFNA17 |
| CIB1 |  |  | IFNA21 |
| CIDEB |  |  | IFNB1 |
| CLU |  |  | IFNG |
| COA8 |  |  | IFNAR1 |
| COL2A1 |  |  | IFNAR2 |
| CRADD |  |  | IFNGR1 |
| CREB3 |  |  | IFNGR2 |
| CREB3L1 |  |  | JAK1 |
| CRH |  |  | JAK2 |
| CRIP1 |  |  | JAK3 |
| CSF2 |  |  | TYK2 |
| CSNK2A1 |  |  | STAT1 |
| CSNK2A2 |  |  | STAT2 |
| CTH |  |  | STAT3 |
| CTNNA1 |  |  | STAT4 |
| CTSC |  |  | STAT5A |
| CTTN |  |  | STAT5B |
| CUL1 |  |  | STAT6 |
| CUL2 |  |  | IRF9 |
| CUL3 |  |  | EIF2AK2 |
| CUL4A |  |  | TLR4 |
| CUL5 |  |  | TICAM2 |
| CX3CL1 |  |  | TICAM1 |
| CX3CR1 |  |  | TLR3 |
| CXCL12 |  |  | ZBP1 |
| CYLD |  |  | USP21 |
| CYP1B1 |  |  | SQSTM1 |
| DAB2IP |  |  | HSP90AA1 |
| DAP |  |  | HSP90AB1 |
| DAP3 |  |  | TNFAIP3 |
| DAPK1 |  |  | PARP1 |
| DAPK2 |  |  | BID |
| DAPK3 |  |  | BAX |
| DAPL1 |  |  | AIFM1 |
| DAXX |  |  | H2AX |
| DBH |  |  | H2AC20 |
| DCC |  |  | H2AC12 |
| DDIAS |  |  | H2AC1 |
| DDIT3 |  |  | H2AW |
| DDIT4 |  |  | H2AB3 |
| DDX3X |  |  | H2AC8 |
| DDX47 |  |  | H2AC4 |
| DDX5 |  |  | MACROH2A2 |
| DEDD |  |  | MACROH2A1 |
| DEDD2 |  |  | H2AC19 |
| DELE1 |  |  | H2AJ |
| DEPTOR |  |  | H2AB1 |
| DIABLO |  |  | H2AC17 |
| DIDO1 |  |  | H2AC18 |
| DNAJA1 |  |  | H2AC11 |
| DNAJC10 |  |  | H2AC21 |
| DNM1L |  |  | H2AZ2 |
| DPF2 |  |  | H2AC7 |
| DYRK2 |  |  | H2AZ1 |
| E2F1 |  |  | H2AC15 |
| E2F2 |  |  | H2AC6 |
| EDA2R |  |  | H2AC13 |
| EIF2AK3 |  |  | H2AC14 |
| ELL3 |  |  | H2AC16 |
| ENO1 |  |  | H2AB2 |
| EP300 |  |  | PPIA |
| EPHA2 |  |  | BCL2 |
| EPO |  |  | TSC1 |
| ERBB3 |  |  | TRIM11 |
| ERCC6 |  |  | IPMK |
| ERN1 |  |  | ITPK1 |
| ERN2 |  |  | SIRT3 |
| ERO1A |  |  | MYC |
| ERP29 |  |  | TNFRSF1B |
| EYA1 |  |  | PANX1 |
| EYA2 |  |  | OTULIN |
| EYA3 |  |  | USP22 |
| EYA4 |  |  | MAP3K7 |
| FADD |  |  | DIABLO |
| FAF1 |  |  | DNMT1 |
| FAIM |  |  | BRAF |
| FAIM2 |  |  | AXL |
| FAM162A |  |  | ID1 |
| FAS |  |  | CDKN2A |
| FASLG |  |  | HSPA4 |
| FASTK |  |  | STUB1 |
| FBH1 |  |  | FLT3 |
| FBXW7 |  |  | HAT1 |
| FEM1B |  |  | SIRT2 |
| FGA |  |  | SIRT1 |
| FGB |  |  | PLK1 |
| FGF10 |  |  | MPG |
| FGFR1 |  |  | BACH2 |
| FGFR3 |  |  | GATA3 |
| FGG |  |  | MYCN |
| FHIT |  |  | ALK |
| FIGNL1 |  |  | ATRX |
| FIS1 |  |  | TERT |
| FNIP2 |  |  | SLC39A7 |
| FXN |  |  | IDH1 |
| FYN |  |  | IDH2 |
| FZD9 |  |  | KLF9 |
| G0S2 |  |  | HDAC9 |
| GABARAP | |  | LEF1 |
| GATA1 |  |  | BNIP3 |
| GATA4 |  |  | CD40 |
| GCLM |  |  | BCL2L11 |
| GDNF |  |  | EGFR |
| GFRAL |  |  | DDX58 |
| GGCT |  |  | TARDBP |
| GHITM |  |  | APP |
| GNAI2 |  |  | TNFRSF21 |
| GNAI3 |  |  |  |
| GPER1 |  |  |  |
| GPX1 |  |  |  |
| GRINA |  |  |  |
| GSDME |  |  |  |
| GSK3A |  |  |  |
| GSK3B |  |  |  |
| GSKIP |  |  |  |
| GSTP1 |  |  |  |
| GZMB |  |  |  |
| HDAC1 |  |  |  |
| HERPUD1 | |  |  |
| HGF |  |  |  |
| HIC1 |  |  |  |
| HIF1A |  |  |  |
| HINT1 |  |  |  |
| HIP1 |  |  |  |
| HIP1R |  |  |  |
| HIPK1 |  |  |  |
| HIPK2 |  |  |  |
| HMGB2 |  |  |  |
| HMOX1 |  |  |  |
| HNRNPK |  |  |  |
| HRAS |  |  |  |
| HRK |  |  |  |
| HSPA1A |  |  |  |
| HSPA1B |  |  |  |
| HSPB1 |  |  |  |
| HTRA2 |  |  |  |
| HTT |  |  |  |
| HYAL2 |  |  |  |
| HYOU1 |  |  |  |
| ICAM1 |  |  |  |
| IFI16 |  |  |  |
| IFI27 |  |  |  |
| IFI27L1 |  |  |  |
| IFI27L2 |  |  |  |
| IFI6 |  |  |  |
| IFNB1 |  |  |  |
| IFNG |  |  |  |
| IGF1 |  |  |  |
| IKBKE |  |  |  |
| IL12A |  |  |  |
| IL19 |  |  |  |
| IL1A |  |  |  |
| IL1B |  |  |  |
| IL2 |  |  |  |
| IL20RA |  |  |  |
| IL33 |  |  |  |
| IL4 |  |  |  |
| IL6R |  |  |  |
| IL7 |  |  |  |
| INCA1 |  |  |  |
| ING2 |  |  |  |
| ING5 |  |  |  |
| INHBA |  |  |  |
| INHBB |  |  |  |
| INS |  |  |  |
| ITGA6 |  |  |  |
| ITGAM |  |  |  |
| ITGAV |  |  |  |
| ITM2C |  |  |  |
| ITPR1 |  |  |  |
| ITPRIP |  |  |  |
| IVNS1ABP | |  |  |
| JAK2 |  |  |  |
| JMY |  |  |  |
| JUN |  |  |  |
| KDM1A |  |  |  |
| KITLG |  |  |  |
| KRT18 |  |  |  |
| KRT8 |  |  |  |
| LCK |  |  |  |
| LGALS12 |  |  |  |
| LGALS3 |  |  |  |
| LRRK2 |  |  |  |
| LTBR |  |  |  |
| LY96 |  |  |  |
| MADD |  |  |  |
| MAEL |  |  |  |
| MAGEA3 |  |  |  |
| MAP2K5 |  |  |  |
| MAP3K5 |  |  |  |
| MAPK7 |  |  |  |
| MAPK8 |  |  |  |
| MAPK8IP1 | |  |  |
| MAPK8IP2 | |  |  |
| MAPK9 |  |  |  |
| MARCHF7 | |  |  |
| MAZ |  |  |  |
| MCL1 |  |  |  |
| MDM2 |  |  |  |
| MELK |  |  |  |
| MFF |  |  |  |
| MIF |  |  |  |
| MIR132 |  |  |  |
| MIR15A |  |  |  |
| MIR16-1 |  |  |  |
| MIR17 |  |  |  |
| MIR198 |  |  |  |
| MIR21 |  |  |  |
| MIR210 |  |  |  |
| MIR221 |  |  |  |
| MIR222 |  |  |  |
| MIR26B |  |  |  |
| MIR27B |  |  |  |
| MIR449A |  |  |  |
| MKNK2 |  |  |  |
| MLH1 |  |  |  |
| MLLT11 |  |  |  |
| MMP9 |  |  |  |
| MNT |  |  |  |
| MOAP1 |  |  |  |
| MPV17L |  |  |  |
| MSH2 |  |  |  |
| MSH6 |  |  |  |
| MSX1 |  |  |  |
| MUC1 |  |  |  |
| MUL1 |  |  |  |
| MYBBP1A | |  |  |
| NACC2 |  |  |  |
| NANOS3 |  |  |  |
| NBN |  |  |  |
| NCK1 |  |  |  |
| NCK2 |  |  |  |
| NDUFA13 |  |  |  |
| NDUFS3 |  |  |  |
| NFATC4 |  |  |  |
| NFE2L2 |  |  |  |
| NGF |  |  |  |
| NGFR |  |  |  |
| NKX3-1 |  |  |  |
| NLE1 |  |  |  |
| NME5 |  |  |  |
| NMT1 |  |  |  |
| NOC2L |  |  |  |
| NOG |  |  |  |
| NOL3 |  |  |  |
| NONO |  |  |  |
| NOS3 |  |  |  |
| NOX1 |  |  |  |
| NR4A2 |  |  |  |
| NUPR1 |  |  |  |
| OPA1 |  |  |  |
| P2RX4 |  |  |  |
| P2RX7 |  |  |  |
| P4HB |  |  |  |
| PAK2 |  |  |  |
| PAK5 |  |  |  |
| PARK7 |  |  |  |
| PARP1 |  |  |  |
| PARP2 |  |  |  |
| PAWR |  |  |  |
| PCGF2 |  |  |  |
| PDCD10 |  |  |  |
| PDCD5 |  |  |  |
| PDCD6 |  |  |  |
| PDIA3 |  |  |  |
| PDK1 |  |  |  |
| PDK2 |  |  |  |
| PDPK1 |  |  |  |
| PDX1 |  |  |  |
| PEA15 |  |  |  |
| PELI3 |  |  |  |
| PERP |  |  |  |
| PF4 |  |  |  |
| PHIP |  |  |  |
| PHLDA3 |  |  |  |
| PIAS4 |  |  |  |
| PIDD1 |  |  |  |
| PIH1D1 |  |  |  |
| PIK3R1 |  |  |  |
| PINK1 |  |  |  |
| PLAGL2 |  |  |  |
| PLAUR |  |  |  |
| PLEKHF1 |  |  |  |
| PLSCR3 |  |  |  |
| PMAIP1 |  |  |  |
| PML |  |  |  |
| POLB |  |  |  |
| POU4F1 |  |  |  |
| POU4F2 |  |  |  |
| PPARD |  |  |  |
| PPIA |  |  |  |
| PPIF |  |  |  |
| PPM1F |  |  |  |
| PPP1CA |  |  |  |
| PPP1R13B | |  |  |
| PPP1R15A | |  |  |
| PPP2R1B |  |  |  |
| PPP3CC |  |  |  |
| PPP3R1 |  |  |  |
| PRDX2 |  |  |  |
| PRELID1 |  |  |  |
| PRKCA |  |  |  |
| PRKCD |  |  |  |
| PRKDC |  |  |  |
| PRKN |  |  |  |
| PRKRA |  |  |  |
| PRODH |  |  |  |
| PSEN1 |  |  |  |
| PSMD10 |  |  |  |
| PSME3 |  |  |  |
| PTEN |  |  |  |
| PTGIS |  |  |  |
| PTH |  |  |  |
| PTPMTI |  |  |  |
| PTPN1 |  |  |  |
| PTPN2 |  |  |  |
| PTPRC |  |  |  |
| PTTGIIP |  |  |  |
| PYCARD |  |  |  |
| QARS1 |  |  |  |
| RACKI |  |  |  |
| RAF1 |  |  |  |
| RB1 |  |  |  |
| RBICC1 |  |  |  |
| RBCK1 |  |  |  |
| RELA |  |  |  |
| RET |  |  |  |
| RFFL |  |  |  |
| RHOTI |  |  |  |
| RHOT2 |  |  |  |
| RIPK1 |  |  |  |
| RIPK3 |  |  |  |
| RNF183 |  |  |  |
| RNF186 |  |  |  |
| RNF34 |  |  |  |
| RNF41 |  |  |  |
| RPL11 |  |  |  |
| RPL26 |  |  |  |
| RPS27L |  |  |  |
| RPS3 |  |  |  |
| RPS6KB1 |  |  |  |
| RPS7 |  |  |  |
| RRPS |  |  |  |
| RTKN2 |  |  |  |
| RTL10 |  |  |  |
| S100A8 |  |  |  |
| MRP14 |  |  |  |
| SCG2 |  |  |  |
| SCN2A |  |  |  |
| SCRT2 |  |  |  |
| SELENOK | |  |  |
| SELENOS |  |  |  |
| SENPI |  |  |  |
| SEPTIN4 |  |  |  |
| SERINC3 |  |  |  |
| SERPINE1 | |  |  |
| SFN |  |  |  |
| SFPQ |  |  |  |
| SFRP1 |  |  |  |
| SFRP2 |  |  |  |
| SGMS1 |  |  |  |
| SGPL1 |  |  |  |
| SGPP1 |  |  |  |
| SH3RF1 |  |  |  |
| SHH |  |  |  |
| SHISA5 |  |  |  |
| SIAHl |  |  |  |
| SIAH2 |  |  |  |
| SIRTI |  |  |  |
| SIVA1 |  |  |  |
| SKIL |  |  |  |
| SLC25A5 |  |  |  |
| SLC35F6 |  |  |  |
| SLC9A3R1 | |  |  |
| SMAD3 |  |  |  |
| SNAII |  |  |  |
| SNAI2 |  |  |  |
| SNWI |  |  |  |
| SOD1 |  |  |  |
| SOD2 |  |  |  |
| SORT1 |  |  |  |
| SP100 |  |  |  |
| SRC |  |  |  |
| SRPX |  |  |  |
| SsT |  |  |  |
| SSTR3 |  |  |  |
| ST20 |  |  |  |
| STK11 |  |  |  |
| STK24 |  |  |  |
| STK25 |  |  |  |
| STK3 |  |  |  |
| STK4 |  |  |  |
| STRADB |  |  |  |
| STX4 |  |  |  |
| STYXL1 |  |  |  |
| SYVNI |  |  |  |
| TAF9 |  |  |  |
| TAF9B |  |  |  |
| TCF7L2 |  |  |  |
| TERT |  |  |  |
| TFDPi |  |  |  |
| TFDP2 |  |  |  |
| TFPT |  |  |  |
| TGFB1 |  |  |  |
| TGFB2 |  |  |  |
| TGFBR1 |  |  |  |
| THBS1 |  |  |  |
| TICAM1 |  |  |  |
| TICAM2 |  |  |  |
| TIMM50 |  |  |  |
| TIMP3 |  |  |  |
| TLR3 |  |  |  |
| TLR4 |  |  |  |
| TM2D1 |  |  |  |
| TMBIM1 |  |  |  |
| TMBIM6 |  |  |  |
| TMC8 |  |  |  |
| TMEM102 | |  |  |
| TMEM109 | |  |  |
| TMEM117 | |  |  |
| TMEM14A | |  |  |
| TMEM161A | |  |  |
| TNF |  |  |  |
| TNFAIP3 |  |  |  |
| TNFRSF10A | |  |  |
| TNFRSF10B | |  |  |
| TNFRSF10C | |  |  |
| TNFRSF12A | |  |  |
| TNFRSF1A | |  |  |
| TNFRSF1B | |  |  |
| TNFRSF25 | |  |  |
| TNFSF10 |  |  |  |
| TNFSF12 |  |  |  |
| TOPORS |  |  |  |
| TP53 |  |  |  |
| TP53BP2 |  |  |  |
| TP63 |  |  |  |
| TP73 |  |  |  |
| TPD52L1 |  |  |  |
| TPT1 |  |  |  |
| TRADD |  |  |  |
| TRAF1 |  |  |  |
| TRAF2 |  |  |  |
| TRAF7 |  |  |  |
| TRAP1 |  |  |  |
| TRIAP1 |  |  |  |
| TRIB3 |  |  |  |
| TRIM32 |  |  |  |
| TRIM39 |  |  |  |
| TXNDC12 | |  |  |
| TYROBP |  |  |  |
| UACA |  |  |  |
| UBB |  |  |  |
| UBE2K |  |  |  |
| UBE4B |  |  |  |
| UBOLN1 |  |  |  |
| UMOD |  |  |  |
| UNC5B |  |  |  |
| URI1 |  |  |  |
| USP28 |  |  |  |
| USP47 |  |  |  |
| VDAC2 |  |  |  |
| VNN1 |  |  |  |
| WDR35 |  |  |  |
| WNT4 |  |  |  |
| WWOX |  |  |  |
| XBP1 |  |  |  |
| YAP1 |  |  |  |
| YBX3 |  |  |  |
| YWHAB |  |  |  |
| YWHAE |  |  |  |
| YWHAG |  |  |  |
| YWHAH |  |  |  |
| YWHAQ |  |  |  |
| YWHAZ |  |  |  |
| ZC3HC1 |  |  |  |
| ZDHHC3 |  |  |  |
| ZMYND11 | |  |  |
| ZNF205 |  |  |  |
| ZNF385A |  |  |  |
| ZNF385B |  |  |  |
| ZNF622 |  |  |  |
| ZSWIM2 |  |  |  |

**Table S3.** Primer sequences used in this study.

| Gene | Forward (5’---3’) | Reverse (5’---3’) |
| --- | --- | --- |
| MRP14 | CCCTGACACCCTGAGCAAGAAG | TGCCATCAGCATCATACACTCCTC |
| IL-1R1 | GGTGGAGGACTCAGGATATTACTATTG | CACAAGCCAGGGTCATTCTCTAAC |
| CXCR2 | GTTCTGCTACGGGTTCACACTG | CAAGGACGACAGCGAAGATGAC |
| CXCR4 | ACGCCACCAACAGTCAGAGG | ATGAAGTCAGGTATAGTCAGGAGGAG |

**Supplementary Figures and Figure Legends**


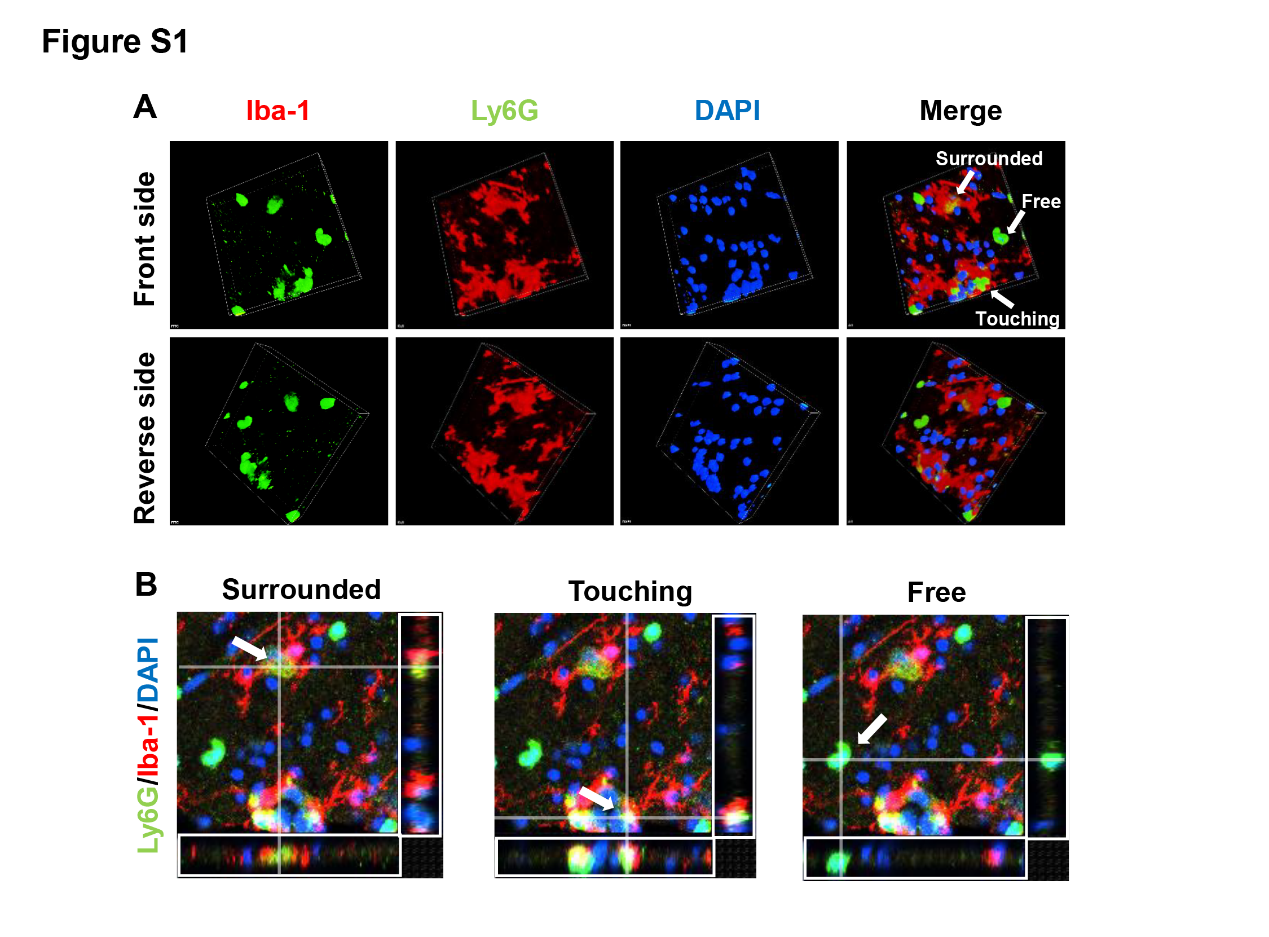


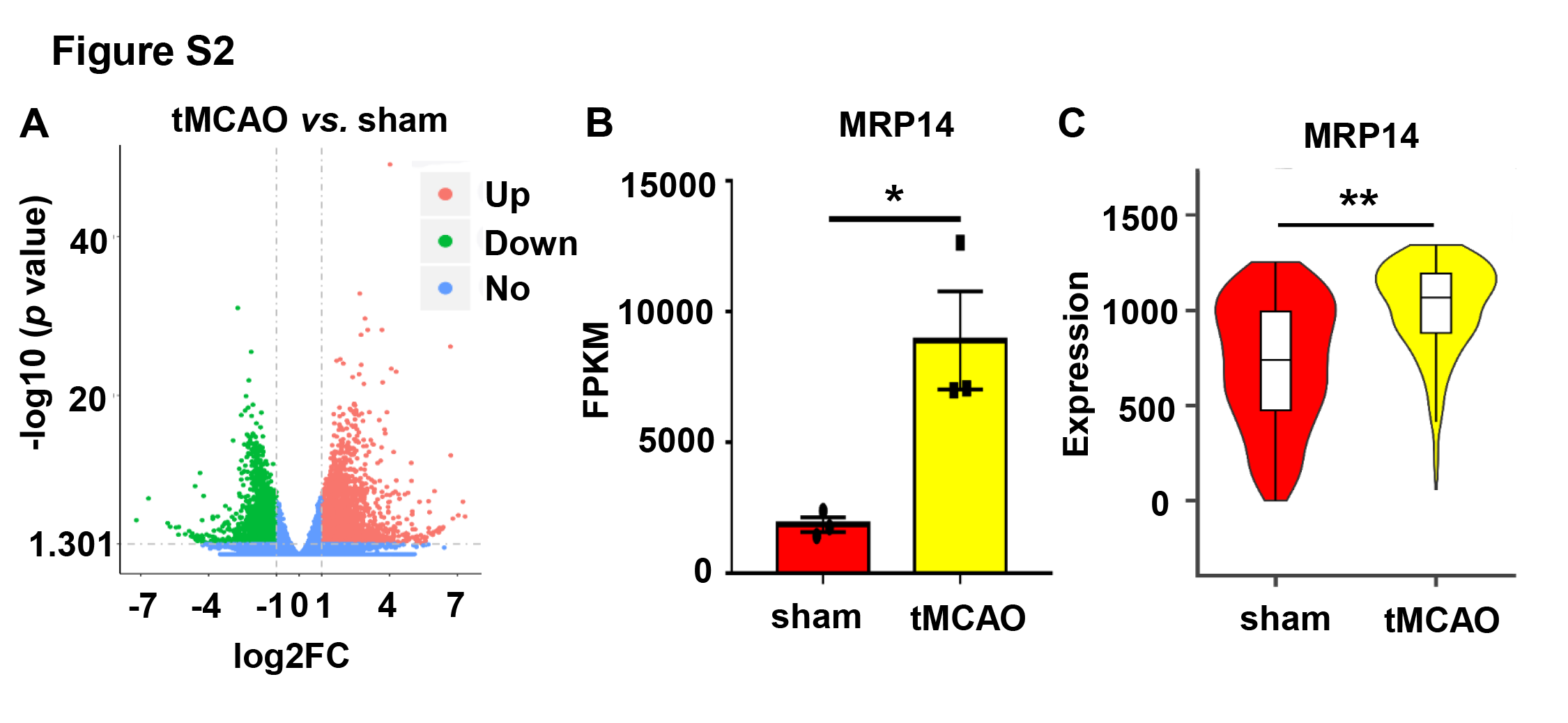

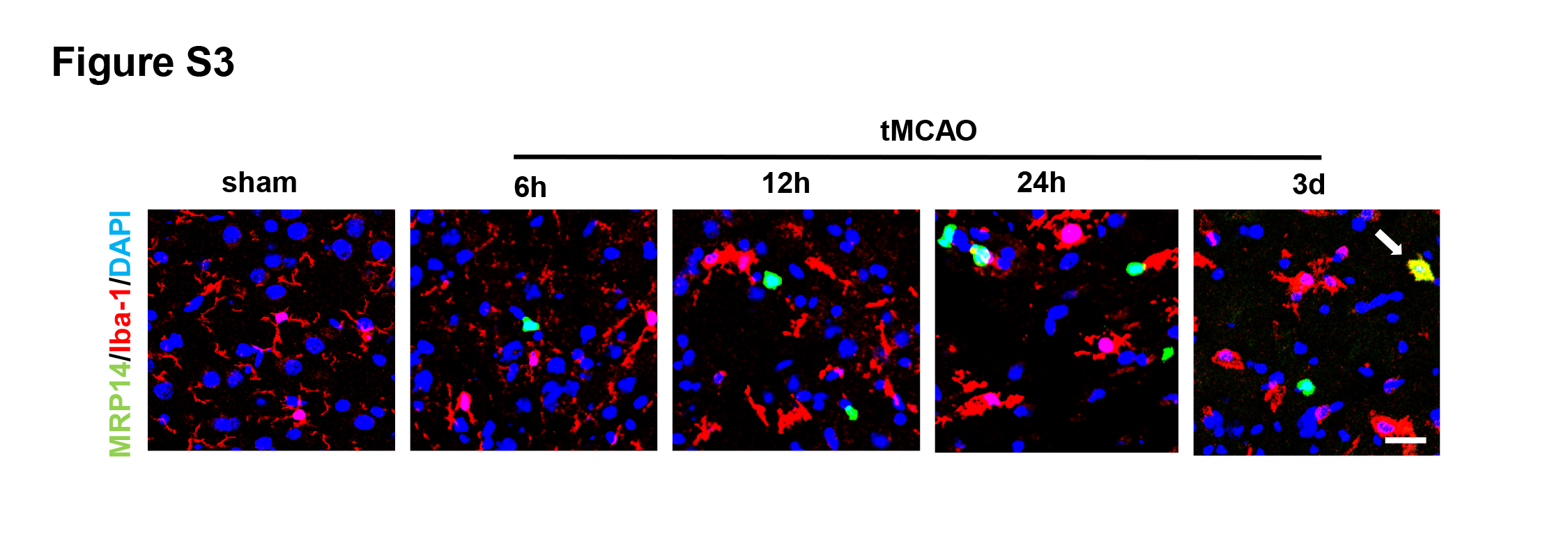

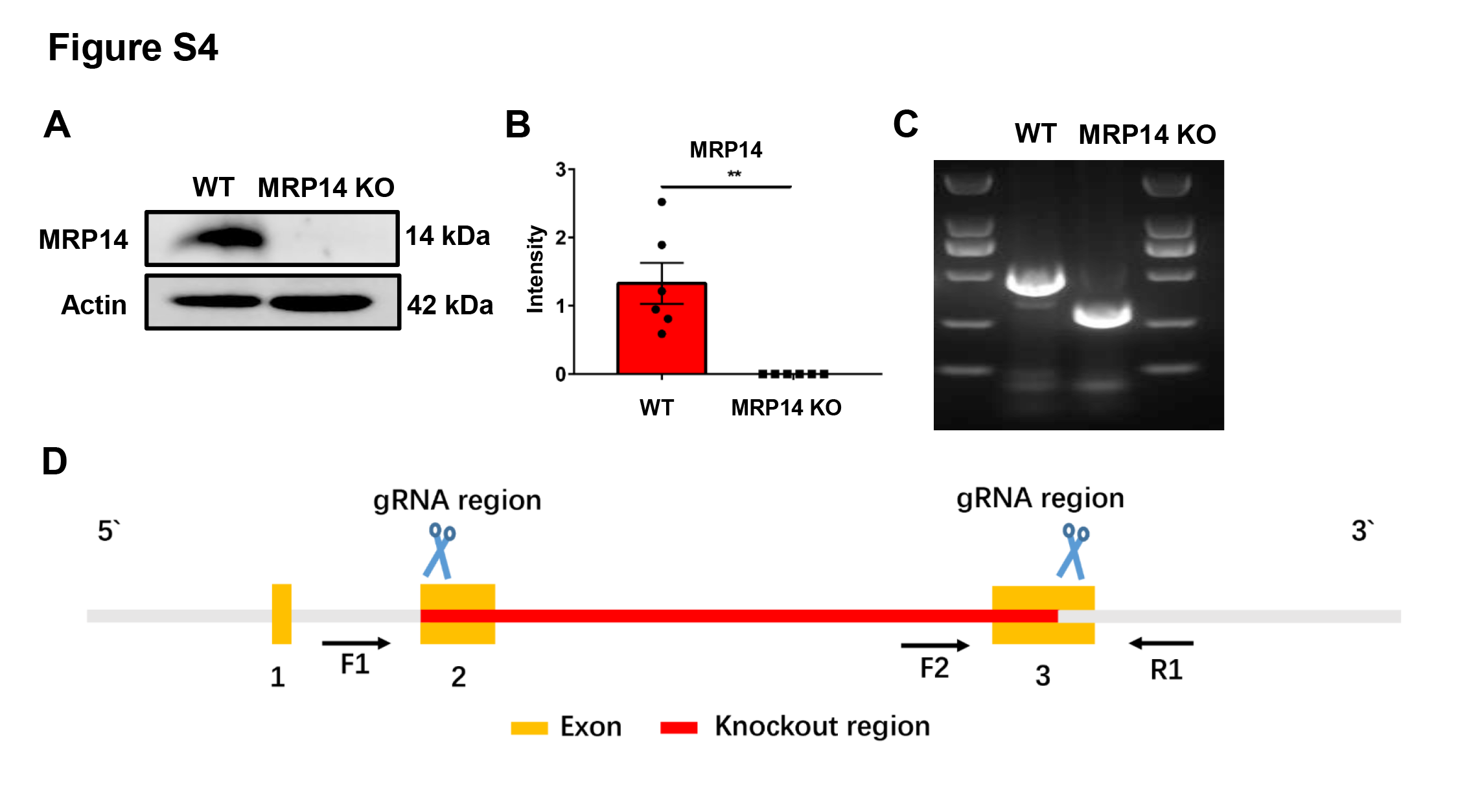

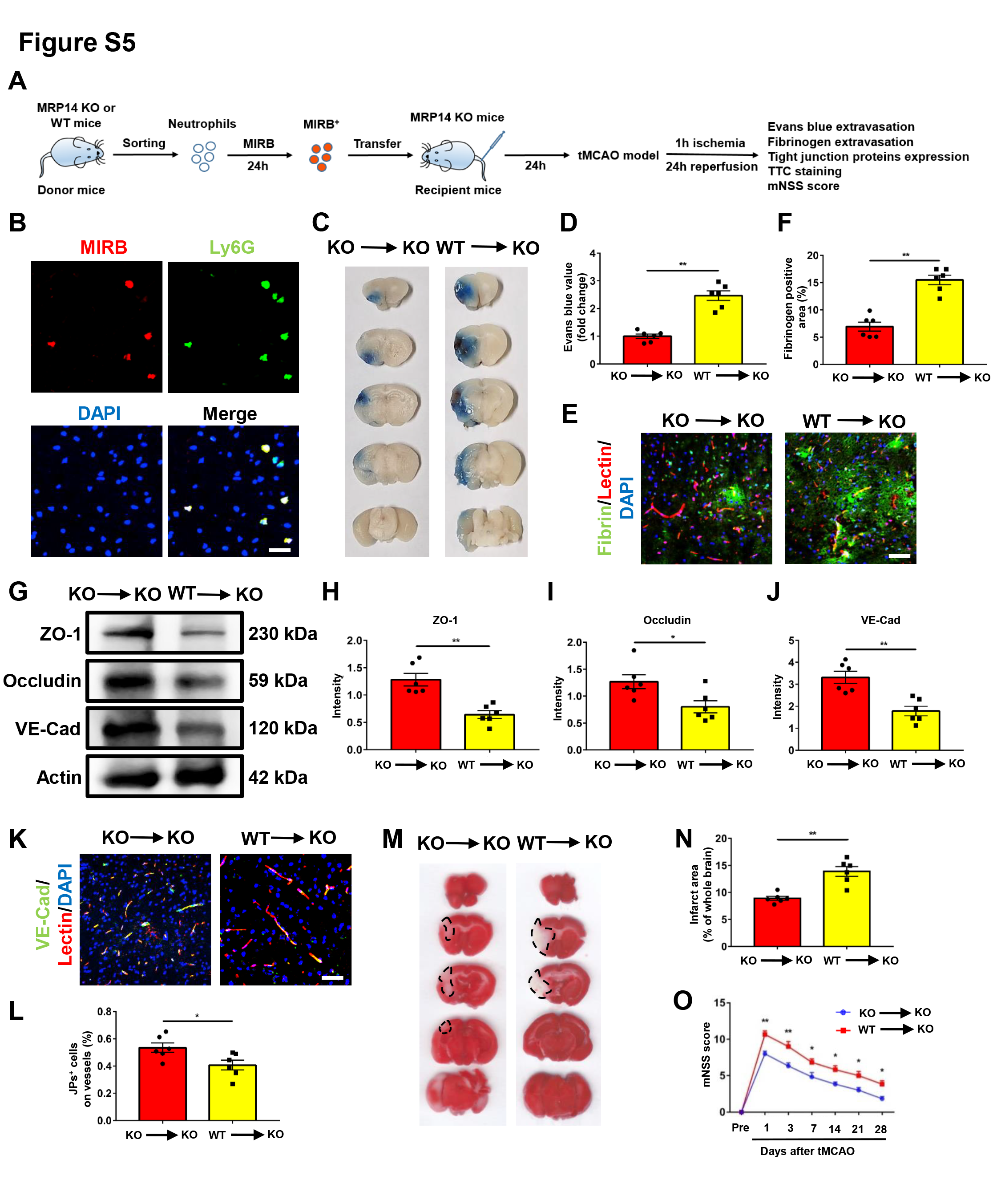

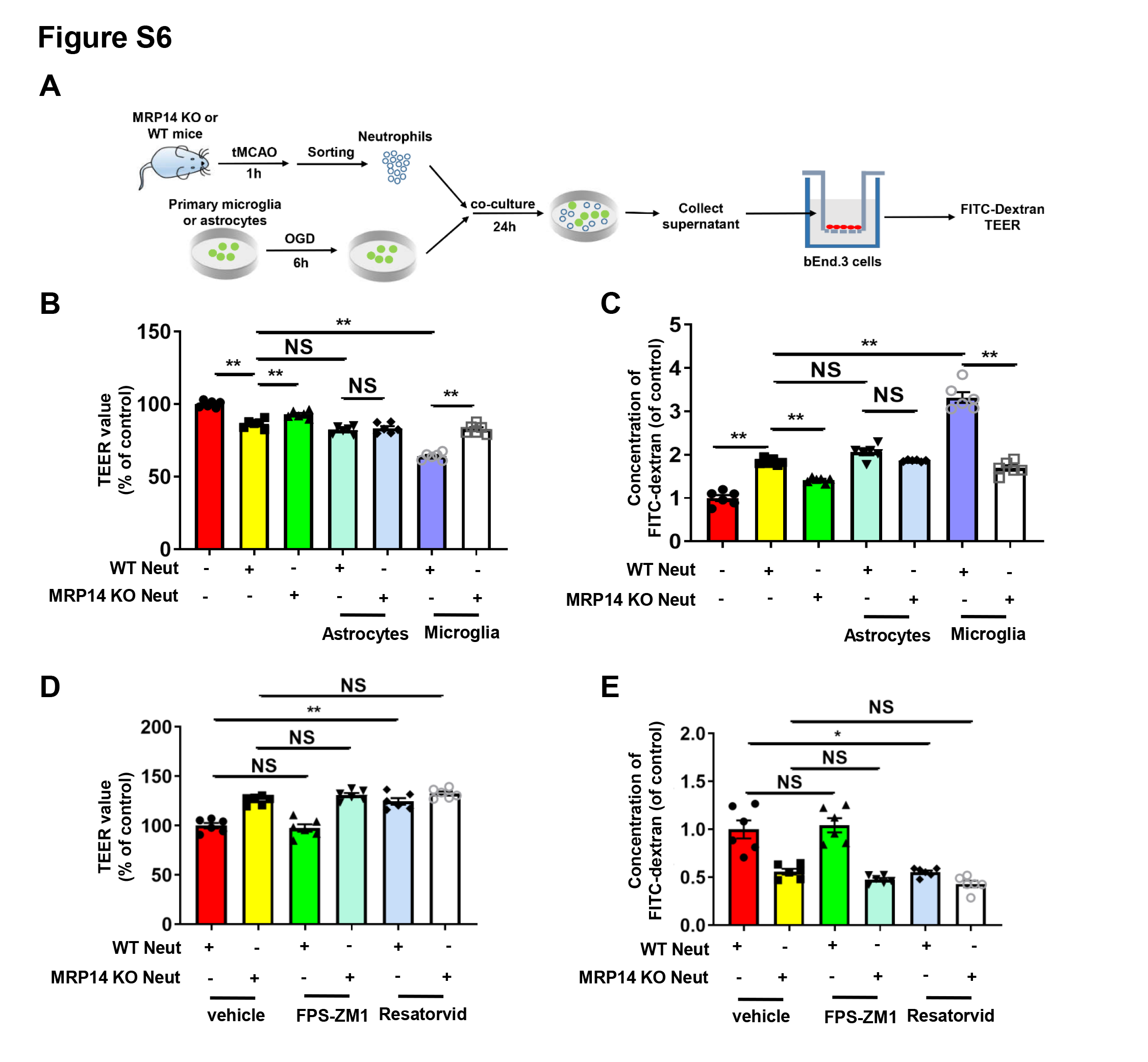

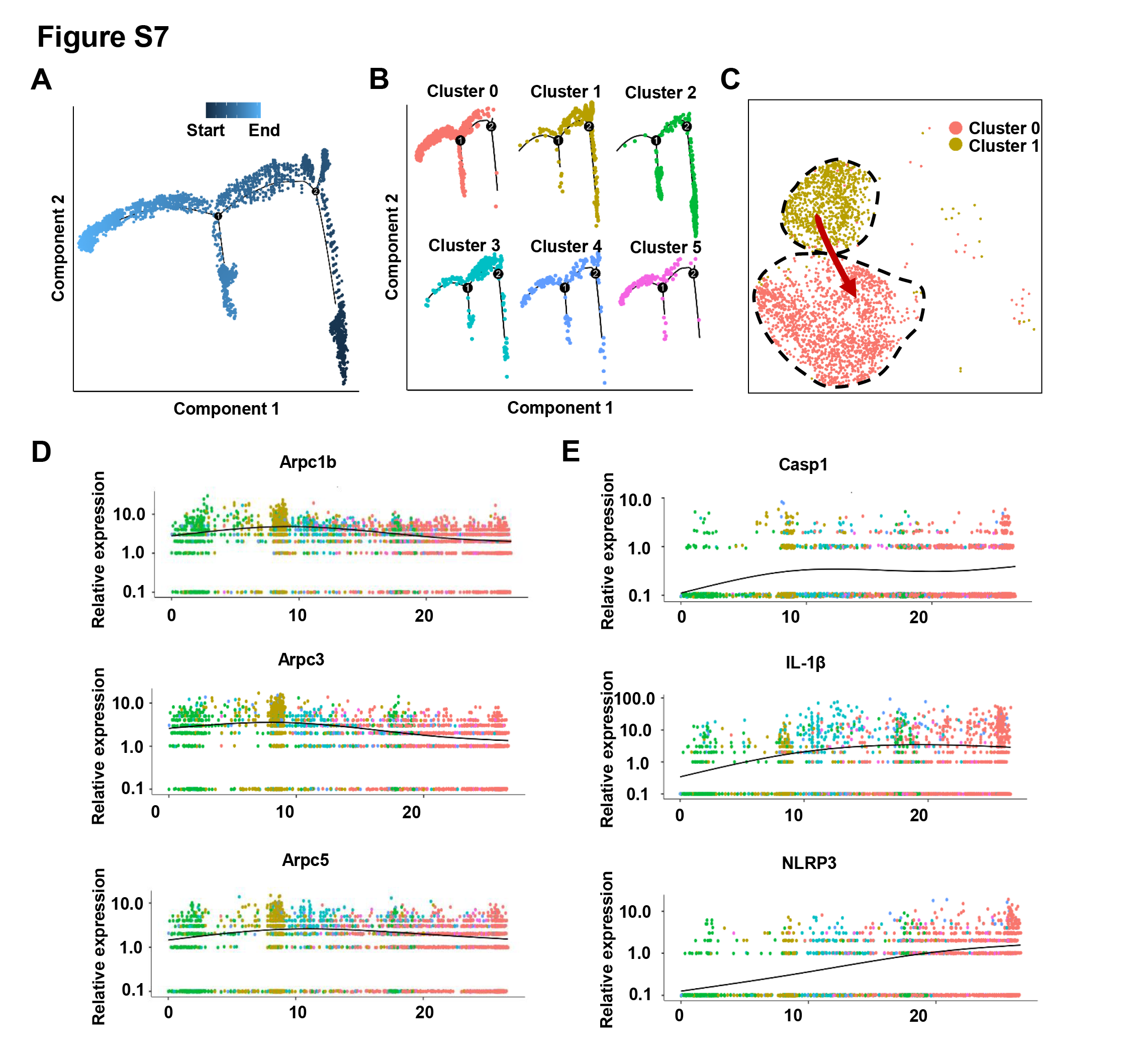

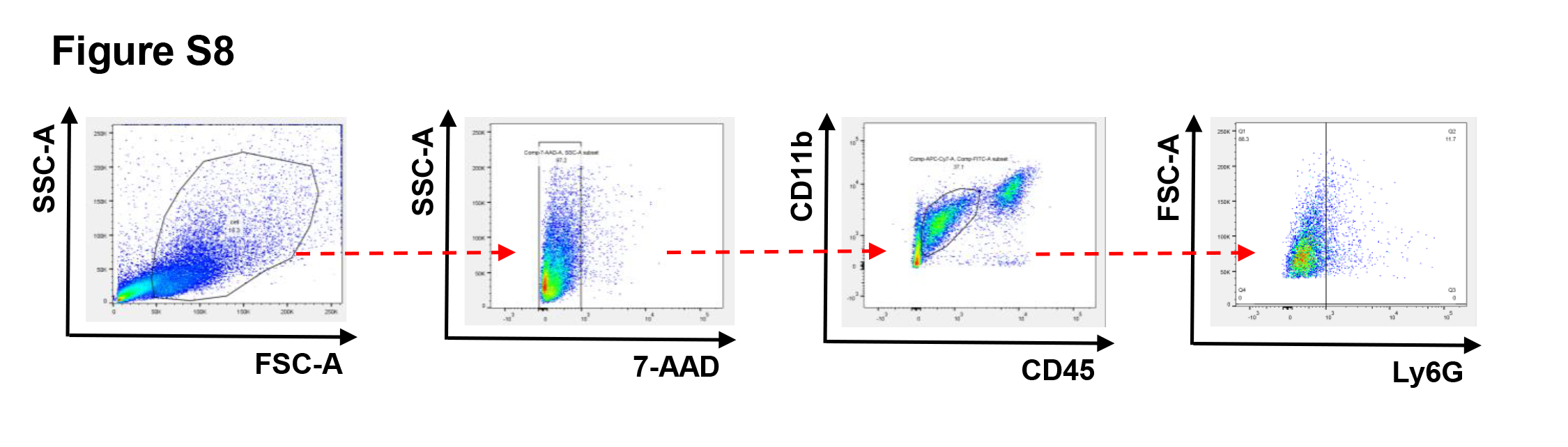

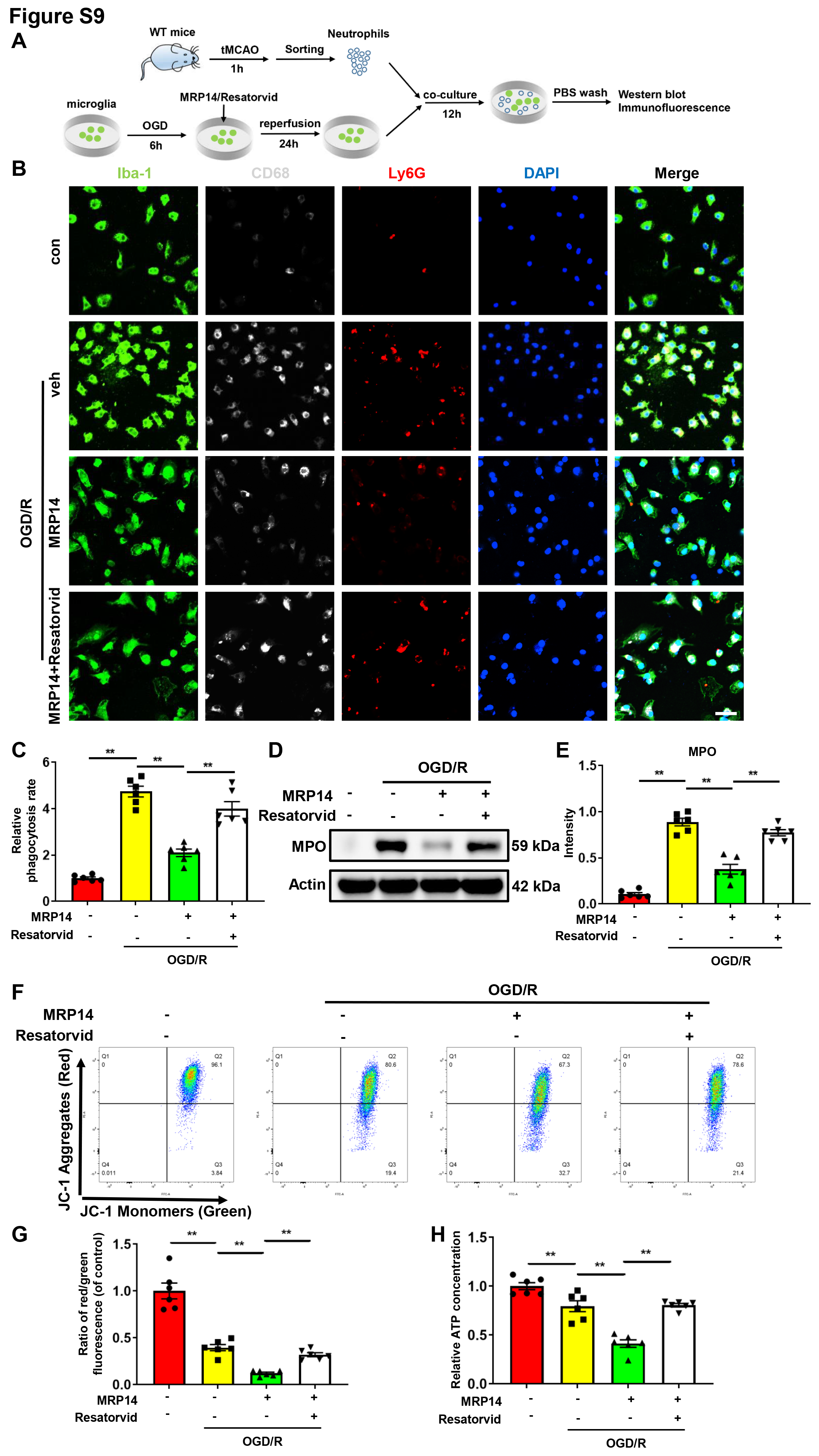

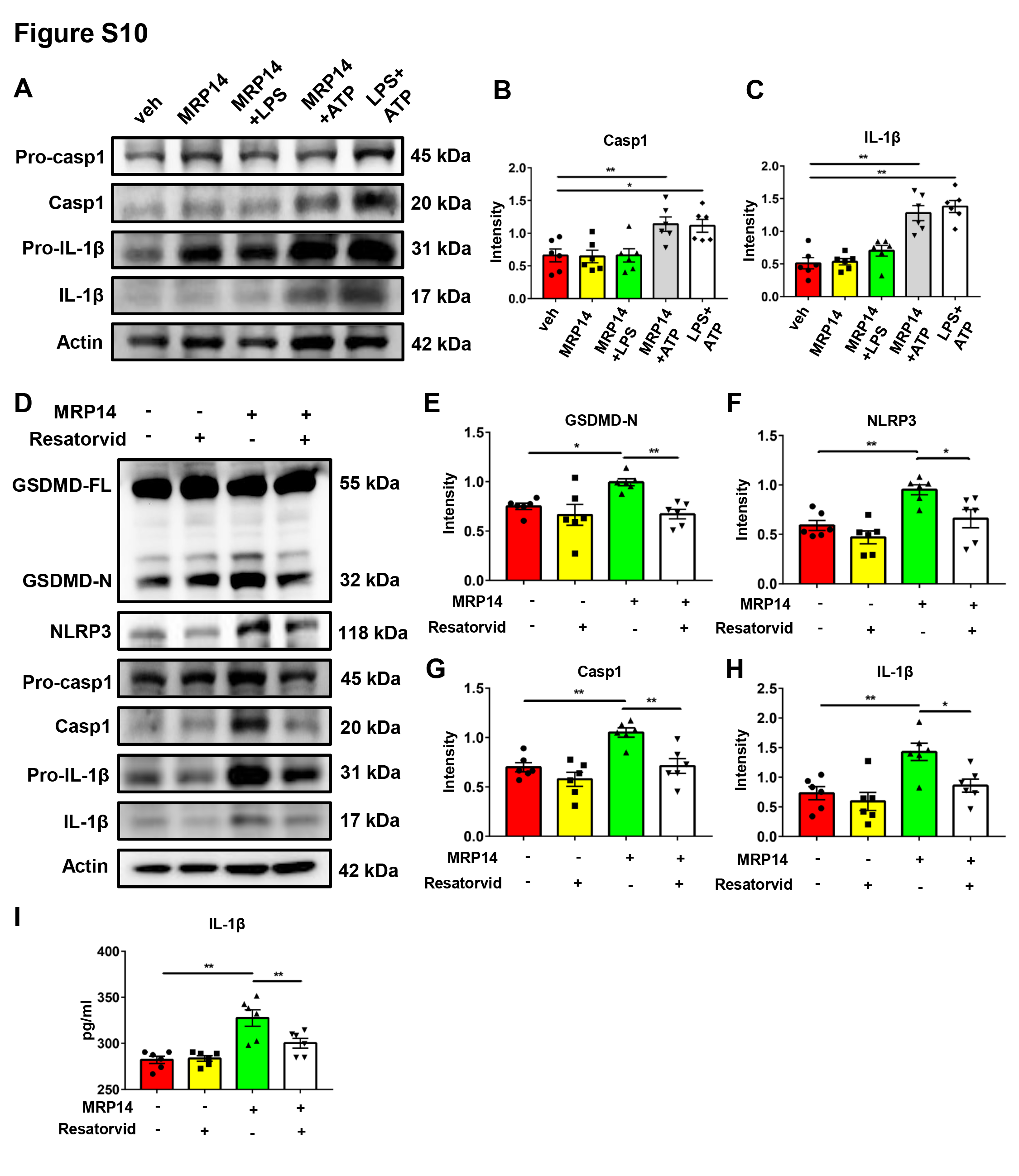

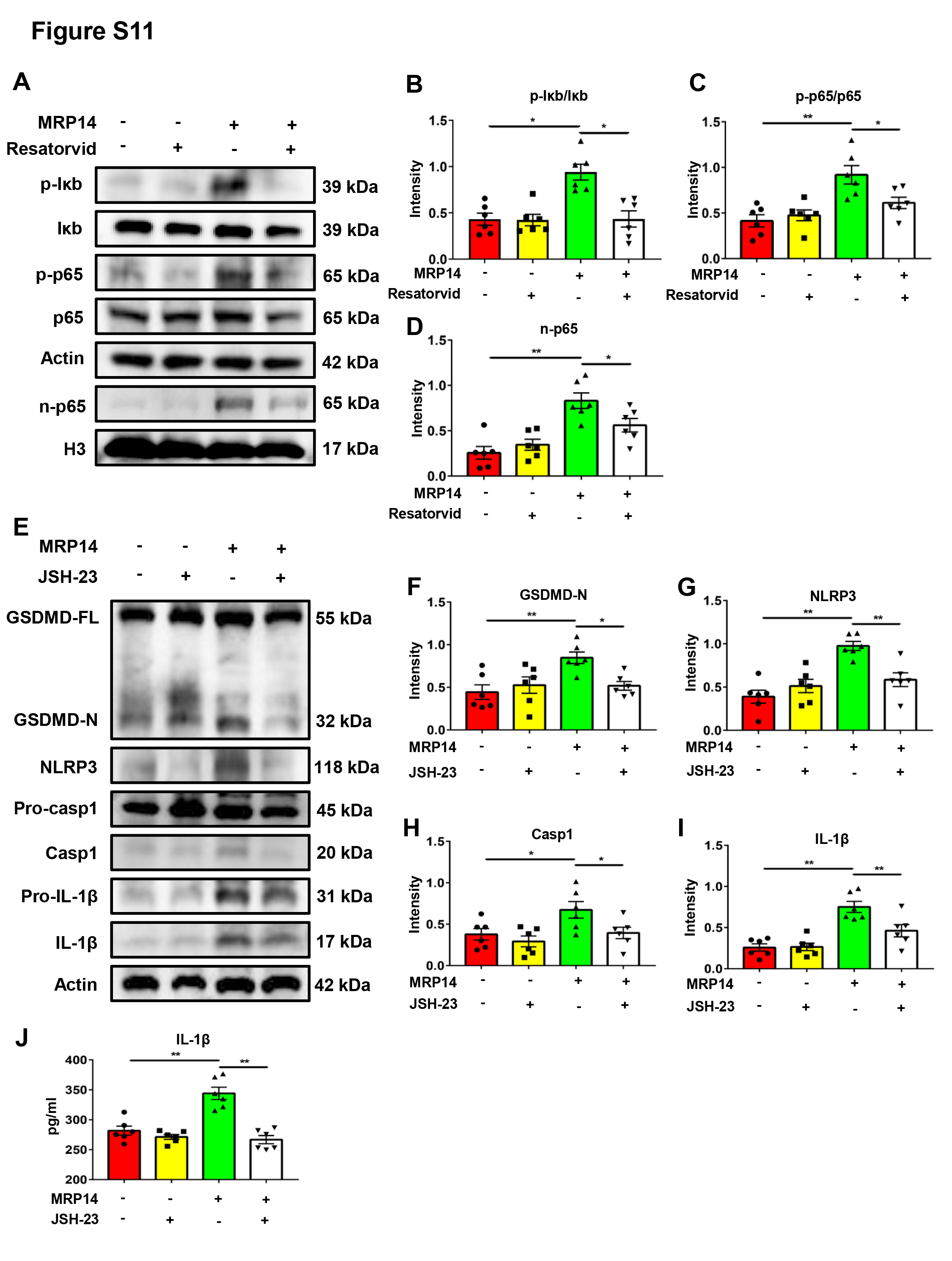

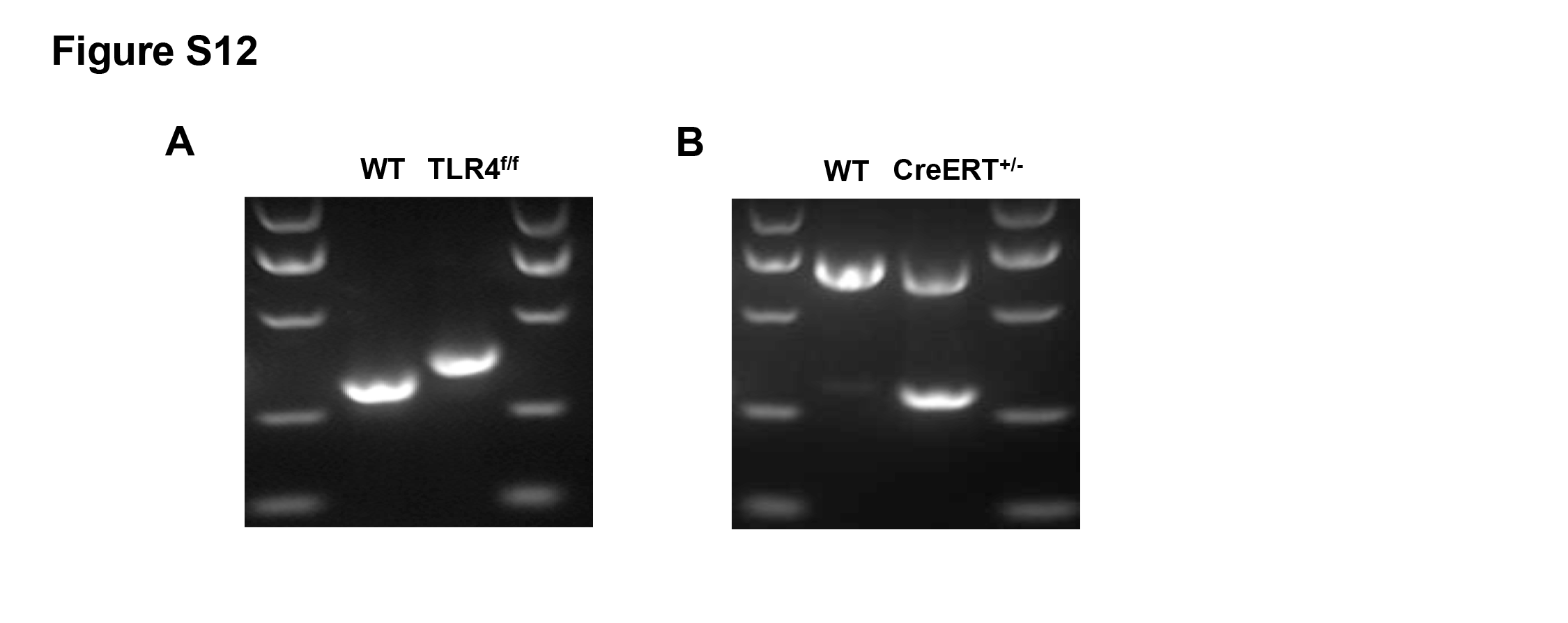

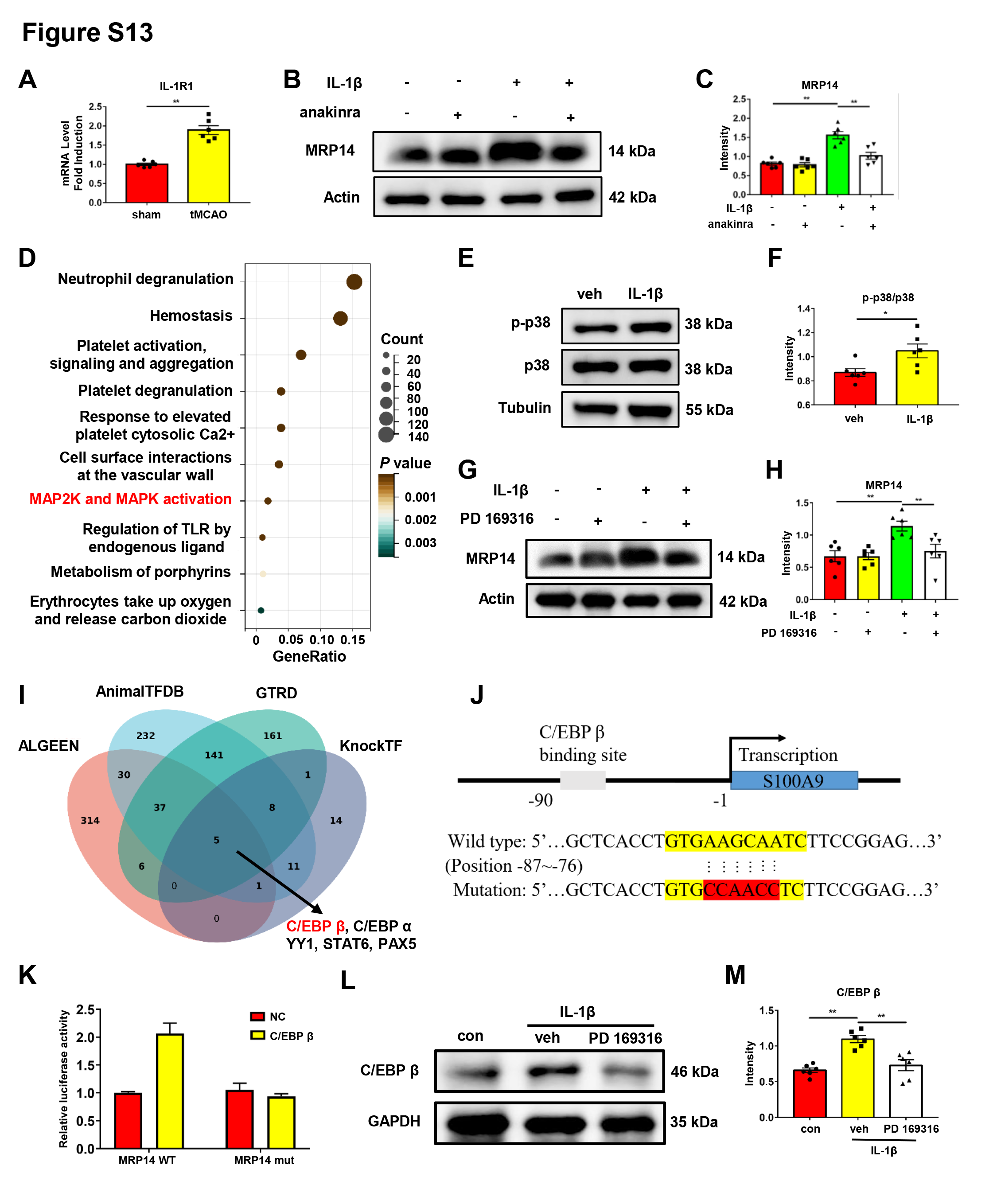

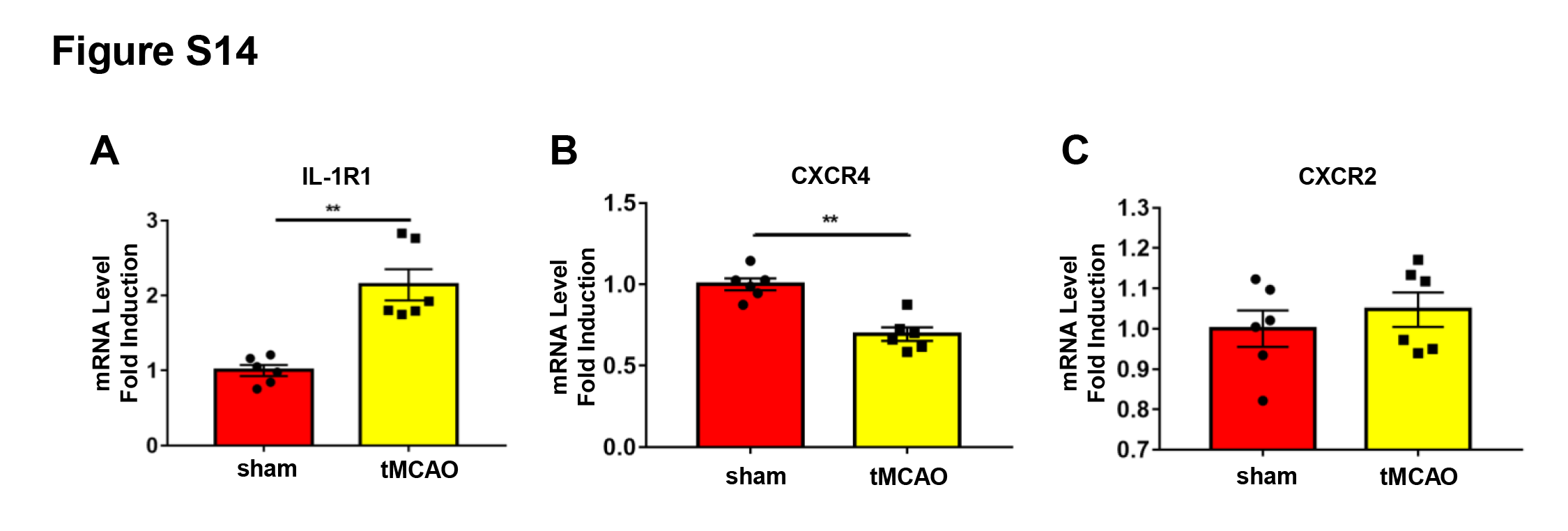


**Figure legend**

**Figure S1: Microglia can engulf neutrophils in the ischemic brain of tMCAO mice.**

A-B. The 3D reconstructions of microscopy images showing the position relationship between Iba-1^+^ microglia and Ly6G^+^ neutrophils in the ischemic brain of tMCAO mice.

**Figure S2: The expression of neutrophil MRP14 in the RNA-sequencing datasets.**

A. The volcano plot showing DEGs of peripheral neutrophils between the sham and tMCAO mice in bulk RNA-sequencing dataset.

B. The expression of neutrophil MRP14 in the peripheral neutrophils from the sham and tMCAO mice in bulk RNA-sequencing dataset.

C. The expression of neutrophil MRP14 in the sorted blood myeloid cells from mice at steady-state and 24 hours after tMCAO in the dataset GSE174440.

Data are presented as means ± SEM; unpaired t-test, Mann-Whitney test. **P* < 0.05, ***P* < 0.01. DEGs, differentially expressed genes; tMCAO, transient middle cerebral artery occlusion; FPKM, fragments per kilobase of exon model per million mapped fragments.

**Figure S3: Immunofluorescence staining showing the MRP14^+^ microglia in the ischemic brain of tMCAO mice at different time points.**

Scale bar: 25 μm. tMCAO, transient middle cerebral artery occlusion.

**Figure S4: The verification of MRP14 knockout in MRP14 KO mice.**

A-B. Western blot analysis of the MRP14 expression in the WT and MRP14 KO mice (n = 6/group).

C-D. Genotyping of MRP14 KO mice.

Data are presented as means ± SEM; unpaired t-test. ***P* < 0.01. KO, knockout.

**Figure S5: Transfer of WT neutrophils into MRP14 KO mice aggravates BBB disruption after ischemic stroke.**

A. The illustration of the experimental schedule.

B. Immunofluorescence staining showing that the MIRB-labeled neutrophils were successfully entered the brain parenchyma of the tMCAO mice.

C-D. The Evans blue extravasation assay of the MRP14 KO mice transferred with WT or MRP14 KO neutrophils (n = 6/group).

E-F. Immunofluorescence staining exhibiting the fibrinogen (green) leakage in the ischemic brain of MRP14 KO mice transferred with WT or MRP14 KO neutrophils (n = 6/group).

G-J. Western blot analysis of the expression of tight junction proteins, including ZO-1, Occludin and VE-Cadherin in the ischemic brain of MRP14 KO mice transferred with WT or MRP14 KO neutrophils (n = 6/group).

K-L. Immunofluorescence staining showing the expression of VE-Cadherin (green) on the blood vessels (red) in the ischemic brain of MRP14 KO mice transferred with WT or MRP14 KO neutrophils (n = 6/group).

M-N. The TTC staining showing the infarction area of the MRP14 KO mice transferred with WT or MRP14 KO neutrophils (n = 6/group).

O. The mNSS scores showing the neurological deficits of the MRP14 KO mice transferred with WT or MRP14 KO neutrophils (n = 6/group).

Data are presented as means ± SEM; Mann-Whitney test, unpaired t-test. **P* < 0.05, ***P* < 0.01. Scale bar: 25 μm (B); 50 μm (E and K). WT, wild type; KO, knockout; BBB, blood brain barrier; MIRB, Molday ION Rhodamine B; tMCAO, transient middle cerebral artery occlusion; TTC, tri-phenyl tetrazolium chloride; mNSS, Modified Neurological Severity Score.

**Figure S6:** **MRP14 could induce more pronounced endothelial cell damage by stimulating microglia.**

A: An illustration of the experimental design.

B-C. The TEER value and leakage of FITC-dextran in the *in vitro* BBB model in the indicated groups (n = 6/group).

D-E. The TEER value and leakage of FITC-dextran in the *in vitro* BBB model in the indicated groups. Microglia were pretreated with TLR4 receptor inhibitor, resatorvid (100 nM) or RAGE receptor inhibitor, FPS-ZM1 (10 μM) (n = 6/group).

Data are presented as means ± SEM; one-way ANOVA. **P* < 0.05, ***P* < 0.01. KO, knockout; BBB, blood brain barrier; TEER, Transendothelial Electric Resistance; FITC, fluorescein isothiocyanate; TLR4, Toll-like receptor 4; RAGE, receptor for advanced glycation endproducts.

**Figure S7: The pseudotime analysis revealed dynamic transformation among microglial clusters.**

A. The distribution of microglial cells along the pseudotime trajectory illustrating the transforming direction.

B. The distribution of each microglial cluster along the pseudotime trajectory.

C. The arrow indicated a directional transition pattern between cluster 0 and cluster 1.

D. The dynamic expression of Arpc1b, Arpc3, and Arpc5 along the pseudotime trajectory.

E. The dynamic expression of caspase 1, IL-1β, and NLRP3 along the pseudotime trajectory.

Arpc, Actin-Related Protein 2/3 Complex; Casp1, caspase 1; IL-1β, interleukin-1β; NLRP3, NOD-like receptor family, pyrin domain containing 3.

**Figure S8: Flow cytometry dot plots exhibit the gating strategy of Ly6G^+^ microglia in ischemic brain.**

**Figure S9: Recombined MRP14 treatment inhibits microglial phagocytosis of neutrophils after OGD/R by causing mitochondrial dysfunction *via* TLR4 receptor *in vitro*.**

A. An illustration of the experimental design.

B-C. Immunofluorescence staining showing the expression of CD68 (white) and phagocytosis of neutrophils (red) by primary microglia (green) in the indicated groups (n = 6/group).

D-E. Western blot analysis of the expression of MPO in primary microglia in the indicated groups (n = 6/group).

F-G. The mitochondrial membrane depolarization in primary microglia in the indicated groups (n = 6/group).

H. The ATP level in primary microglia in the indicated groups (n = 6/group).

Data are presented as means ± SEM; one-way ANOVA. ***P* < 0.01. Scale bar: 25 μm. OGD/R, oxygen-glucose deprivation/reperfusion; TLR4, Toll-like receptor 4; MPO, myeloperoxidase; WT, wild type; BM, bone marrow; PBS, phosphate buffer solution; ATP, adenosine triphosphate.

**Figure S10**: **MRP14 promotes microglial pyroptosis and NLRP3 inflammasome activation *via* TLR4 *in vitro*.**

A-C. Western blot analysis of the expression of pro-caspase 1, caspase 1, pro-IL-1β and IL-1β in the primary microglia treated with vehicle, MRP14, MRP14 + LPS, MRP14 + ATP or LPS + ATP. After 6 hours of OGD, the primary microglia were stimulated with recombined MRP14 protein (1 μg/ml) for 24 hours, followed by the treatment of 2.5 mM ATP for 30 minutes (n = 6/group). The LPS was added at 500 ng/ml 6 hours before recombined MRP14 or ATP treatment.

D-H. Western blot analysis of the expression of GSDMD, NLRP3, pro-caspase 1, caspase 1, pro-IL-1β and IL-1β in the OGD/R-stimulated primary microglia treated with MRP14 and resatorvid (n = 6/group).

I. The ELISA test of IL-1β level in the supernatant of OGD/R-stimulated primary microglia treated with MRP14 and resatorvid (n = 6/group).

Data are presented as means ± SEM; Kruskal-Wallis test, one-way ANOVA. **P* < 0.05, ***P* < 0.01. NLRP3, NOD-like receptor family, pyrin domain containing 3; TLR4, Toll-like receptor 4; LPS, lipopolysaccharide; ATP, adenosine triphosphate; GSDMD, gasdermin D; IL-1β, interleukin-1β; Casp1, caspase 1; ELISA, enzyme-linked immunosorbent assay.

**Figure S11: MRP14 regulates microglial NLRP3 inflammasome-associated pyroptosis through TLR4/NF-κB signaling pathway *in vitro*.**

A through J, the OGD/R-stimulated primary microglia were treated with recombined MRP14 protein at a dose of 1 μg/ml for 24 hours, followed by the treatment of 2.5 mM ATP for 30 minutes. Resatorvid and JSH-23 were added into the microglial culture at 100 nM and 10 μM, respectively, 1 hour before recombined MRP14 treatment.

A-D. Western blot analysis of the expression of p-Iκb, Iκb, p-p65, p65 and nuclear p65 in the primary microglia treated with MRP14 and Resatorvid (n = 6/group).

E-I. Western blot analysis of the expression of GSDMD, NLRP3, pro-caspase 1, caspase 1, pro-IL-1β and IL-1β in the primary microglia treated with MRP14 and NF-κB inhibitor JSH-23 (n = 6/group).

J. The ELISA test of IL-1β level in the supernatant of primary microglia treated with MRP14 and JSH-23 (n = 6/group).

Data are presented as means ± SEM; one-way ANOVA. **P* < 0.05, ***P* < 0.01. NLRP3, NOD-like receptor family, pyrin domain containing 3; TLR4, Toll-like receptor 4; ATP, adenosine triphosphate; GSDMD, gasdermin D; Casp1, caspase 1; IL-1β, interleukin-1β; n-p65, nuclear p65; ELISA, enzyme-linked immunosorbent assay.

**Figure S12: Genotyping of TLR4^flox/flox^ and Cre-ERT^+/-^ mice.**

A. Genotyping of TLR4^flox/flox^ mice.

B. Genotyping of Cre-ERT^+/-^ mice.

TLR4, Toll-like receptor 4.

**Figure S13: IL-1β promotes neutrophil MRP14 expression *via* the IL-1R1/P38/C/EBP β signaling.**

A-C and E-M, Neutrophils isolated from the peripheral blood of tMCAO mice 1 hours after ischemia were treated with recombined IL-1β at 10 ng/ml for 4 hours, and anakinra (10 µg/ml) or PD 169316 (10 μM) was added 30 minutes before IL-1β treatment.

A. The mRNA level of IL-1R1 in the peripheral neutrophils of the sham and tMCAO groups (n = 6/group).

B-C. Western blot analysis of the MRP14 expression in peripheral neutrophils treated with IL-1β and IL-1R1 inhibitor anakinra (n = 6/group).

D. The REACTOME pathway enrichment analysis of the upregulated DEGs of peripheral neutrophils between the tMCAO and sham groups in previous RNA-sequencing.

E-F. Western blot analysis of the expression of p-p38 and p38 in the IL-1β treated neutrophils (n = 6/group).

G-H. Western blot analysis of the expression of MRP14 in neutrophils treated with IL-1β and p38 inhibitor PD 169316 (n = 6/group).

I. The Venn diagram among the predicted transcription factors of MRP14 by database of ALGEEN, GTRD and AnimalTFDB and the enriched transcription factors of the DEGs in the RNA-sequencing analysis by database KnockTF.

J-K. The dual luciferase assay in 293T cells co-transfected with C/EBP β plasmids and PGL3.basic plasmids with wild-type MRP14 promoter, or mutant sequence (n = 6/group).

L-M. Western blot analysis of the C/EBP β expression in neutrophils treated with vehicle, IL-1β or IL-1β + PD 169316 (n = 6/group).

Data are presented as means ± SEM; unpaired t-test, one-way ANOVA, two-way ANOVA. **P* < 0.05, ***P* < 0.01. IL-1β, interleukin-1β; tMCAO, transient middle cerebral artery occlusion; DEGs, differentially expressed genes.

**Figure S14: The expression of IL-1R1 in the bone marrow neutrophils is elevated in the tMCAO mice.**

A-C. The mRNA levels of IL-1R1, CXCR4 and CXCR2 in the bone marrow neutrophils isolated from the sham and tMCAO mice.

Data are presented as means ± SEM; Mann-Whitney test, unpaired t-test. ***P* < 0.01.
